# Increased prolactin levels in pregnancy affect colorectal cancer aggressiveness

**DOI:** 10.1186/s12915-025-02500-8

**Published:** 2026-01-07

**Authors:** Maria Lopez-Cavestany, Olivia A. Wright, Alexandria T. Carter, Brittany O’Brian, Cathy Eng, Michael R. King

**Affiliations:** 1 Department of Biomedical Engineering, Vanderbilt University, Nashville, TN 37212 USA; 2 Department of Bioengineering, Rice University, Houston, TX 77030 USA; 3 Division of Hematology Oncology, Vanderbilt University Medical Center, Nashville, TN 37212 USA

**Keywords:** Colorectal cancer, Pregnancy, Cancer aggressiveness, Computational modeling, Liposomal therapies

## Abstract

**Background:**

Colorectal cancer (CRC) is diagnosed during approximately 1 in 13,000 pregnancies and is associated with worse outcomes, including a higher incidence of metastatic disease at diagnosis and reduced maternal survival compared to non-pregnant patients. In this study, we investigated two key contributors to this phenomenon: (1) the increased cancer aggressiveness driven by elevated prolactin (PRL) levels during pregnancy and (2) the limited treatment options available to pregnant CRC patients.

**Results:**

For the first time, we demonstrate that pregnancy-level PRL directly enhances JAK2/STAT3 and JAG1/NOTCH1 signaling in CRC cells, promoting epithelial-mesenchymal transition (EMT) and cancer stem-like protein expression. We developed and fitted a computational model of the JAK2/STAT3 signaling pathway to our in vitro data, identifying specific nodes within the cascade that are most sensitive to PRL fluctuations during pregnancy. Clinically, we highlight data from CRC cases at Vanderbilt University Medical Center, which underscore the more advanced stage at diagnosis in pregnant patients and the limited treatment options available due to concerns about fetal safety. Additionally, we show that PRL exposure sensitizes CRC cells to TRAIL-induced apoptosis, supporting the potential of TRAIL-based therapies, particularly in liposomal form, as a pregnancy-compatible treatment approach.

**Conclusions:**

This study provides the first mechanistic link between pregnancy-level prolactin and increased CRC aggressiveness through JAK2/STAT3 and JAG1/NOTCH1 signaling. It also suggests a novel therapeutic direction by demonstrating that PRL sensitizes CRC cells to TRAIL-induced apoptosis. Together, our findings highlight the need for new therapeutic strategies for safe and effective treatment of CRC in pregnant patients.

**Resumen:**

El cáncer colorrectal (CRC) se diagnostica durante aproximadamente 1 de cada 13.000 embarazos y se asocia con peores pronósticos, incluyendo una mayor incidencia de metastásis en el momento del diagnóstico y una menor supervivencia en comparación con pacientes no embarazadas. En este estudio investigamos dos factores clave que contribuyen a este fenómeno: (1) el aumento de la agresividad de las células cancerígenas causado por los niveles elevados de prolactina (PRL) durante el embarazo, y (2) las limitaciones de las opciones terapéuticas disponibles para pacientes embarazadas con CRC.

**Resultados:**

Por primera vez demostramos que los niveles de PRL durante el embarazo aumentan la señalización JAK2/STAT3 y JAG1/NOTCH1 en células de CRC, incrementando la transición epitelio-mesénquima (EMT) y la expresión de proteínas asociadas a un fenotipo de células madre cancerosas. Desarrollamos y ajustamos un modelo in silico de la vía de señalización JAK2/STAT3 basado en nuestros datos in vitro, identificando nodos específicos dentro de la cascada que son especialmente sensibles a las fluctuaciones de PRL durante el embarazo. Clínicamente, destacamos datos de casos de CRC del Vanderbilt University Medical Center, que muestran un estadío más avanzado en el diagnóstico en pacientes embarazadas y las opciones terapéuticas restringidas debido a preocupaciones sobre la seguridad fetal. Además, mostramos que la exposición a PRL sensibiliza a las células de CRC a la apoptosis inducida por TRAIL, lo que respalda el potencial de terapias basadas en TRAIL, particularmente en liposomas, como un enfoque terapéutico compatible con el embarazo.

**Conclusiones:**

Este estudio proporciona el primer vínculo mecanístico entre los niveles de prolactina durante el embarazo y el aumento de la agresividad del CRC a través de la señalización JAK2/STAT3 y JAG1/NOTCH1. También sugerimos una nueva dirección terapéutica al demostrar que la PRL sensibiliza las células de CRC a la apoptosis inducida por TRAIL. En conjunto, el estudio subraya la necesidad de nuevas estrategias terapéuticas para el tratamiento seguro y eficaz del CRC en pacientes embarazadas.

**Supplementary Information:**

The online version contains supplementary material available at 10.1186/s12915-025-02500-8.

## Background

During pregnancy, 1 in 13,000 women is diagnosed with colorectal cancer (CRC), a number that is predicted to increase in coming years [1]. Two primary factors are expected to contribute to this rise in cases. First, the incidence rate of CRC has more than doubled since 1980 in young adults [2–4]. Since the median age for CRC in pregnancy is 32, the number of these cases will likely increase due to CRC case numbers increasing in this age group [1]. The second factor that influences this trend is the increase in women choosing to delay pregnancy until later in life [5, 6]. CRC risk increases with age, and with the average age of first pregnancy increasing worldwide, the incidence of CRC during pregnancy will likely increase in the future [7]. Diagnosing CRC during pregnancy presents challenges, often leading to delays that allow the disease to progress. A major reason for this is overlapping common symptoms such as rectal bleeding, abdominal pain, and constipation [1]. These delays in CRC diagnoses of pregnant cases provide critical time for the disease to progress. At the time of diagnosis, 73.2% of CRC cases in pregnant women have reached an advanced stage; in comparison, 50% of age-matched non-pregnant women cases have reached an advanced stage [8, 9]. Metastases are identified in approximately 48% of pregnant CRC patients at the time of diagnosis [1]. One study compiled 119 pregnant patient case reports and found the median maternal survival to be 36 months post-diagnosis [1]. The high morbidity of pregnant CRC patients demonstrates the need for increased understanding of how pregnancy impacts CRC progression.

During pregnancy, the female body undergoes substantial physiological change, yet the underlying consequences of these adaptations on the pathology of CRC are largely unknown. Prolactin (PRL) is a pleotropic hormone that is overexpressed in pregnant women, with pregnant women having PRL serum levels as high as 60 ng/mL in early pregnancy and 120 ng/mL in late pregnancy, compared to 20 ng/mL PRL serum levels for non-pregnant women [10]. PRL plays a significant role in the lactation and reproduction processes of mammals by acting on a cellular level to affect growth, development, metabolism, and immunoregulation [11]. PRL causes physiological changes by activating signaling pathways when it binds to the PRL receptor (PRLR). The principal signaling pathway activated by PRL-PRLR interactions is the janus kinase (JAK)/signal transducer and activator of transcription (STAT) pathway, specifically the JAK2/STAT3 pathway. Under healthy conditions, this molecular pathway translates extracellular cytokine signaling into intracellular signals that modulate cellular immune processes, proliferation, differentiation, survival, and development [12]. Opportunistic cancer cells are known to take advantage of aberrant JAK/STAT signaling to increase oncogenic activity [13].

Previous clinical research has shown that CRC patients with hyperprolactinemia, or serum concentration of PRL over 20 ng/mL, had worse prognosis and shorter overall survival compared to patients with healthy PRL serum levels [14]. In parallel, PRL has been detected in tumor samples from CRC patients, where increased expression correlated with more differentiated tumors, suggesting paracrine/autocrine signaling activity [15]. There have also been several studies which link elevated PRL levels with cancer progression and increasing cancer stage [16, 17]. Additionally, a study by *Neradugomma *et al*.* in 2014 showed that CRC patient tumor samples and several CRC cell lines overexpress PRLR compared to healthy colon tissue [18]. The expression of PRLR has also been confirmed in the CRC cell lines HCT116, HT29, SW480, SW620, and DLD1. When CRC cells were treated with PRL in vitro, increased STAT3 phosphorylation occurred as quickly as 1 min after treatment, leading to increased production of Jagged1 (JAG1) mRNA. JAG1 binds to NOTCH1 receptors on neighboring cells leading to a cleaving event where the NOTCH1 intracellular cleaved domain (NICD) activates the transcription of stem cell-associated proteins such as CD44 and CD133 as well as genes involved in cell growth, differentiation, survival, and tumorigenesis [19, 20].

Treatment options for pregnant colorectal cancer patients are complex, as both the maternal and neonatal health must be considered. Within the first trimester, surgical intervention and chemotherapy are possible, but as the pregnancy continues, they become more dangerous to fetal health [21]. Tumor-necrosis factor-related apoptosis-inducing ligand (TRAIL) is an anticancer therapy that selectively induces apoptosis in cancer cells by binding to death receptors on the cell membrane [22, 23]. All TRAIL agents, however, remain in stage I due to their low efficacy in unbound form [24]. Further in vitro and in vivo studies have shown that in its liposomal form, TRAIL is a much more effective anticancer drug [25]. Additionally, it can be used to treat cancer cells that are metastasizing through the bloodstream (circulating tumor cells or CTCs) to help prevent disease progression [26]. Given the high incidence of metastatic disease in pregnant CRC patients, therapies which prevent cancer dissemination by targeting CTCs are of particular interest [1]. Additionally, TRAIL therapy has little to no apoptotic effects on the cells in the gestational and fetal membranes, making it a candidate for treating cancer during pregnancy [27].

**Fig. 1:**
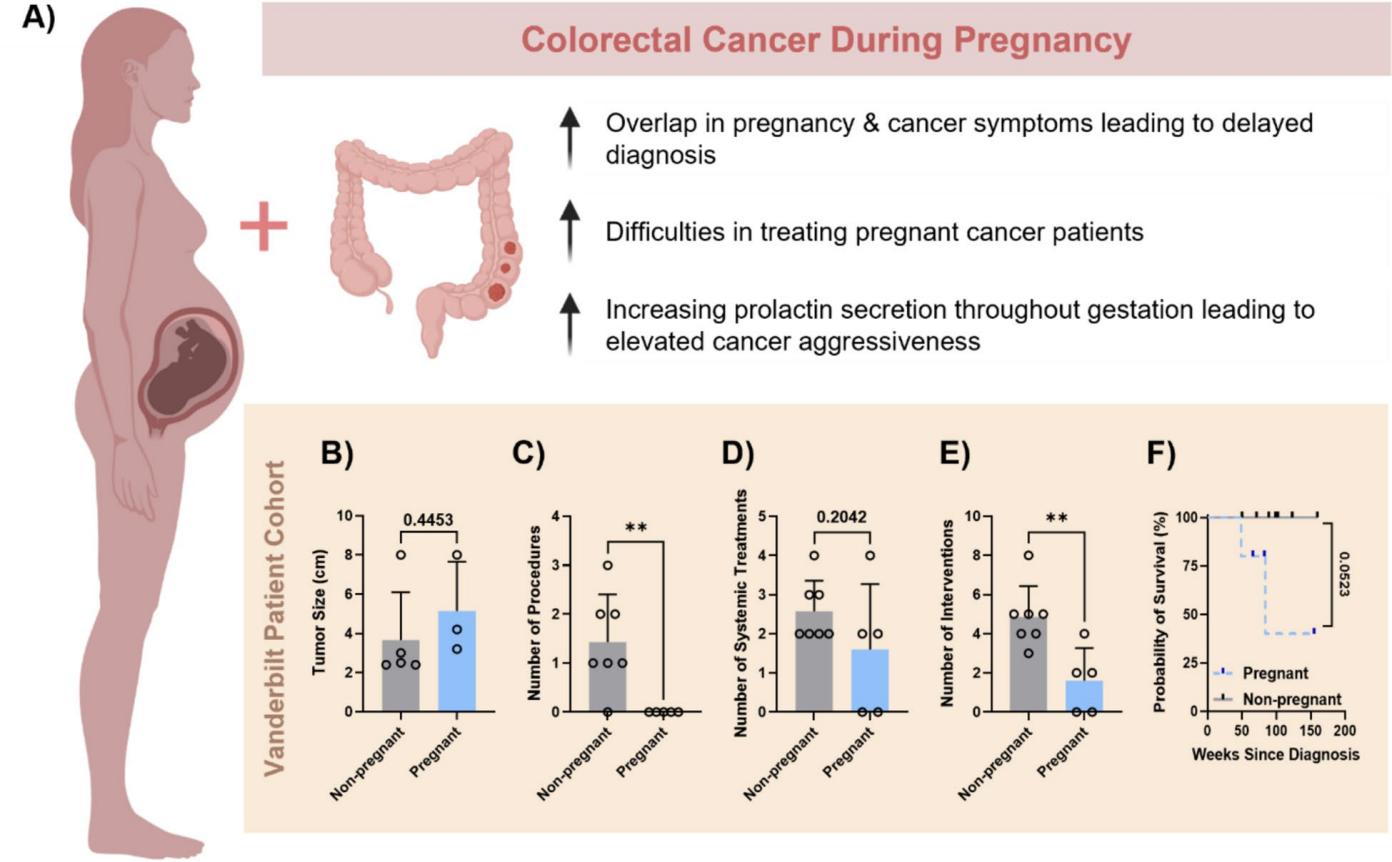
Pregnant colorectal cancer patients have larger tumors yet fewer options exist for anticancer interventions compared to non-pregnant colorectal cancer patients.** A** Overview of factors that lead to increased cancer aggressiveness during pregnancy. Results from pregnant and age-/stage-matched non-pregnant CRC patients at Vanderbilt University Medical Center for **B** tumor size in centimeter at the time of resection, **C** number of procedures, **D** number of systemic treatments, **E** total number of interventions, and **F** probability of survival since date of diagnosis

Throughout this study, we highlight two of the three main factors that likely contribute to the increased aggressiveness of CRC in pregnant patients (Fig. 1A). The first factor of interest is the link between increased prolactin signaling during pregnancy leading to increased cancer cell aggressiveness. We utilized in vitro and computational evidence to explore this effect. In vitro data was then used to link pregnancy levels of PRL to increased JAK2/STAT3 and JAG1/NOTCH1 signaling resulting in increased epithelial-to-mesenchymal transition (EMT) and cancer cell stem-like protein expression. A computational model of the JAK2/STAT3 signaling cascade was built and fitted to the new in vitro data to identify which parts of the signaling cascade were most sensitive to changes in PRL during pregnancy. The second factor is the lack of treatment options for pregnant patients compared to their non-pregnant counterparts [28]. This is highlighted by clinical data from patient cases at Vanderbilt University Medical Center. Additionally, we demonstrated that pregnancy levels of PRL sensitize cancer cells to TRAIL-mediated apoptosis and explore the therapeutic effects of immunotherapy-functionalized liposomes to eliminate circulating tumor cells in the vasculature as a potential method to prevent metastasis in this patient population. A Spanish translation of the abstract is provided in Additional File 1.

## Results

### Pregnant colorectal cancer patients have larger tumors, yet fewer options exist for anticancer interventions compared to non-pregnant colorectal cancer patients

To investigate the clinical impact of pregnancy on the progression and treatment of CRC, we studied the cases of 12 early-onset CRC patients at Vanderbilt University Medical Center, 5 of which were pregnant at the time of diagnosis. The sample size was small given to the rarity of this patient population. The study was reviewed and approved by the Vanderbilt University Institutional Review Board. Early-onset colorectal cancer is defined as those diagnosed under 45; therefore, for the purpose of this study, only women between the ages of 25–45 years old were considered. The clinicopathological data gathered from the patients were the following: patient demographic data, medical history, physician/staff notes concerning cancer diagnosis, diagnostic evaluations including cancer treatment history, disease status, laboratory values including molecular profiling, radiographic studies, pathology reports, surgery reports, radiation reports, drug administration records, and clinical outcomes (response to treatment, side effects, progression or relapse-free survival, overall survival, etc.). The patient demographics are summarized in Table 1.

**Table 1:** Summary of pregnant and age-/stage-matched non-pregnant CRC patients at Vanderbilt University Medical Center

Pt	Age	Status^a^	Trimester^b^	Tumor location^c^	Tumor size^d^	Cancer stage			Interventions
						T	N	M	
***Pregnant***									
1	41	A	1	R	–	3b	1	–	*Systemic*: 5-FU, oxaliplatin
2	35	A	1	R	4.2	3	1	–	No interventions
3	33	D	3	R	8	4b	2	–	*Systemic*: 5-FU, oxaliplatin
4	22	A	Delivery	CR	3.2	4a	2b	1b	No interventions
5	41	D	–	CL	–	4a	1	–	*Systemic*: Biologics, 5-FU, oxaliplatin, panitumumab
***Not pregnant***									
6	28	A	–	CL	8	4	1b	–	*Procedures*: Hemicolectomy *Systemic*: 5-FU, oxaliplatin
7	40	A	–	CL	2.5	3	1a	–	*Procedures*: Partial colectomy *Systemic*: 5-FU, oxaliplatin, irinotecan
8	35	A	–	R	2.4	1	–	–	*Procedure*: Low anterior resection *Systemic*: 5-FU, oxaliplatin
9	36	A	–	CL	2.4	3	2b	–	*Procedures*: Hemicolectomy, right hepatectomy Systemic: 5-FU
10	30	A	–	CL	–	3	1a	–	*Procedures*: Partial colectomy, left hepatectomy *Systemic*: 5-FU, oxaliplatin, irinotecan
11	42	A	–	CL	3	3	1c	–	*Procedures*: Partial colectomy, exploratory laparotomy, small Intestine resection *Systemic*: 5-FU, oxaliplatin, bevacizumab
12	–	A	–	CL	–	4	–	–	*Systemic*: 5-FU, oxaliplatin, irinotecan

^a^Status as of 2024, where A is alive and D is deceased.

^b^Trimester at diagnosis.

^c^Tumor location, where R is rectum, CR is right-side colon, and CL is left-side colon.

^d^Tumor size at resection in cm

In the patient cohort studied, the average age for the pregnant and non-pregnant CRC patients considered was 34 and 35, respectively. Out of the full patient population studied, nine women were white, two were black, and one was Asian. Within the pregnant patient group, one woman was in her 1st pregnancy, one in her 2nd pregnancy, and three were in their 3rd pregnancy at the time of CRC diagnosis. Additionally, diagnosis occurred within the 1st trimester for two out of the five pregnant patients, and one was diagnosed during delivery. Interestingly, 60% of the pregnant patients had a primary tumor located in the rectum. The other two pregnant patients had left-sided and right-sided tumors. Across all patients in the study, 86% of the non-pregnant patients had left-sided colon cancer. Cancer stage was advanced, and cancer grade investigation found that all tumors were moderately differentiated (except for one in the non-pregnant group which was poorly differentiated). Histology reports showed that all of the tumors from both the pregnant and non-pregnant patients were adenocarcinomas.

Tumors were measured at resection, and, interestingly, there was a trend towards increased tumor size in the pregnant CRC patients compared to the non-pregnant control group (Fig. 1B). On average, women who were pregnant had an average tumor size of 5.1 cm ± 2.5 cm (mean ± SD). In contrast, the non-pregnant patient group had an average tumor size of 3.6 cm ± 2.4 cm (mean ± SD). Additionally, there was a large difference in the number of interventions between the pregnant and non-pregnant CRC patient groups. The term “procedures” encompassed surgical treatment options for the patients. As seen in Fig. 1C, non-pregnant patients had significantly more procedures done compared to the pregnant patient group. All but one non-pregnant patient had a surgical procedure done during their treatment cycle in the medical center. The most common treatments of this type were hemicolectomies and partial colectomies. In comparison, no pregnant patients received this type of treatment. The term “systemic treatments” encompassed chemotherapies and immunotherapies. There was no difference in the number of systemic treatments between the two patient groups (Fig. 1D). The most common chemotherapies used were 5-FU and oxaliplatin. Interestingly, immunotherapies were used in two cases of left-sided CRC: one in a pregnant patient and the other in a non-pregnant patient. Overall, counting both the surgical procedures and systemic treatments, the non-pregnant group still received significantly more interventions than the pregnant patient group (Fig. 1E). The non-pregnant group received on average five total interventions per patient, while the pregnant group received an average of approximately two total interventions per patient. At the beginning of 2024, 3 out of the 5 pregnant CRC patients were still alive (Fig. 1F). In comparison, all CRC patients in the non-pregnant group were alive at the beginning of 2024 and had completed their treatment cycles.

### Pregnancy prolactin levels cause biphasic STAT3 phosphorylation and changes in downstream marker expression

**Fig. 2:**
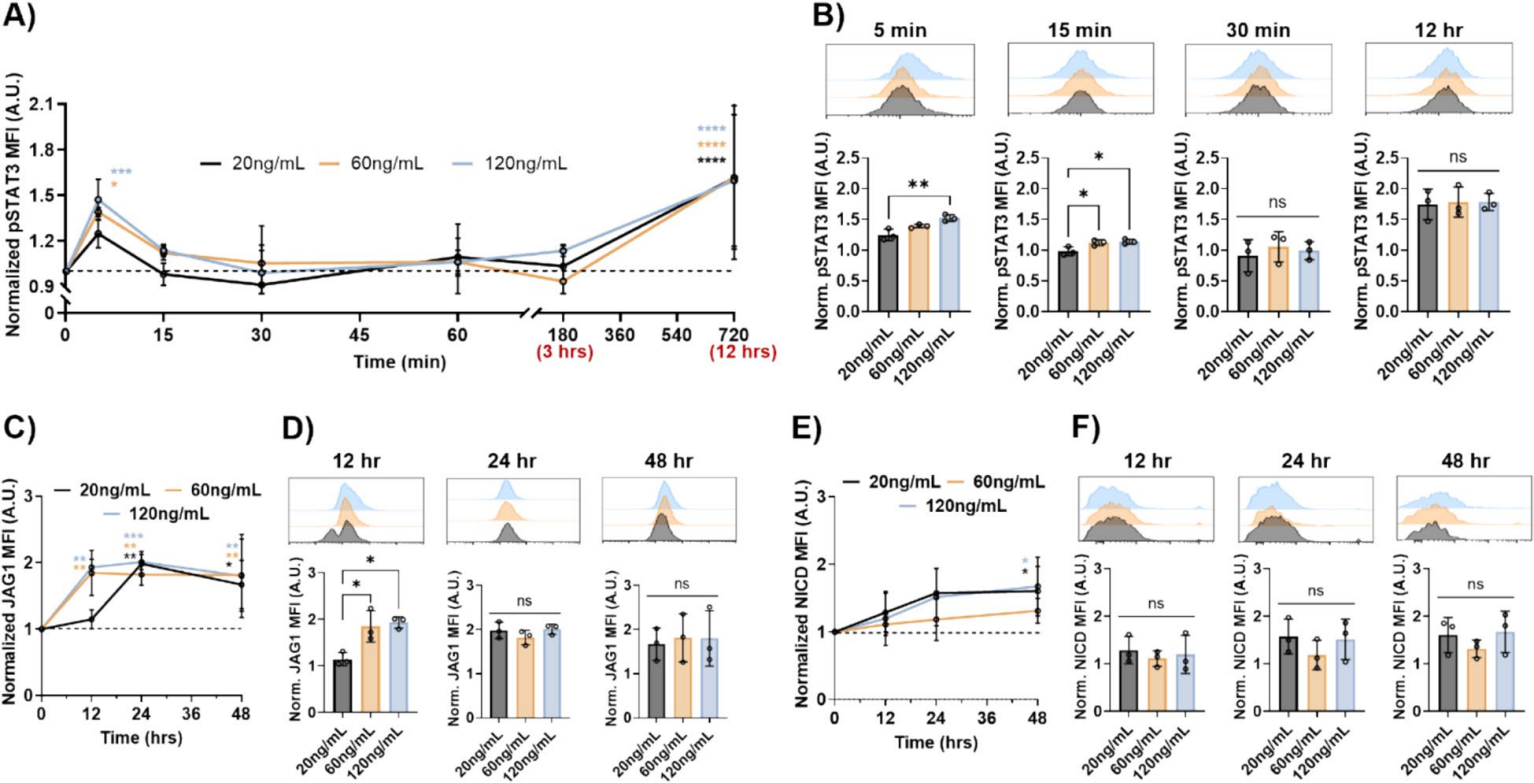
Pregnancy prolactin levels cause biphasic STAT3 phosphorylation and increased JAG1 and NICD expression. **A** XY plot of pSTAT3 expression from 0 to 12 h treated with 20 ng/mL, 60 ng/mL, and 120 ng/mL of PRL. **B** FC histogram and quantification of pSTAT3 expression at 5 min, 15 min, 30 min, and 12 h post-treatment. **C** XY plot of JAG1 expression from 0 to 48 h treated with 20 ng/mL, 60 ng/mL, and 120 ng/mL of PRL. **D** FC histogram and quantification of JAG1 expression at 12, 24, and 48 h post-treatment. **E** XY plot of NICD expression from 0 to 48 h treated with 20 ng/mL, 60 ng/mL, and 120 ng/mL of PRL. **F** FC histogram and quantification of NICD expression at 12, 24, and 48 h post-treatment. Statistical significance was evaluated by two-way ANOVA or ordinary one-way ANOVA and is shown as *p < 0.05, **p < 0.01, ***p < 0.001, and ****p < 0.0001 (*n* = 3 biological replicates)

For all the in vitro experimentation, the colorectal adenocarcinoma cell lines HT29 and COLO320 were utilized as both are derived from female patients. Cells were treated with three different PRL concentrations to reflect healthy, non-pregnant (20 ng/mL), early pregnancy (60 ng/mL), and late pregnancy (120 ng/mL) [10]. A fourth group was left with no treatment to evaluate baseline levels of each protein. First, the STAT3 phosphorylation was evaluated at a short timescale and up until 12 h after treatment [29]. After 5 min, STAT3 phosphorylation in the HT29 cells in both the 60 ng/mL and the 120 ng/mL treatment increased significantly compared to the baseline (Fig. 2A). The HT29 cells treated with 20 ng/mL showed a small but not significant increase at this timepoint. At the 5-min timepoint, the cells treated with 120 ng/mL had significantly higher STAT3 phosphorylation than those treated with 20 ng/mL (Fig. 2B). At 15 min, STAT3 phosphorylation had decreased in all three groups for the HT29 cells. Here, both the 60 ng/mL and 120 ng/mL treated cells had small but significantly higher STAT3 phosphorylation compared to the 20 ng/mL treated cells. The STAT3 phosphorylation returned to baseline for all treatment groups at the 30 min mark and was maintained until 3 h post-treatment. No significant differences were observed between the three treatment groups at these timepoints. When the HT29 cells were tested at 12 h after treatment, STAT3 phosphorylation increased significantly by 60% in all treatment groups. No significant differences were observed between treatments at 12 h. The COLO320 cells showed a significant increase in STAT3 phosphorylation with all treatment groups at 5 min, similar to the HT29 cells (Additional File 2: Fig. S1A and B). Interestingly, the COLO320 cells lacked the classic biphasic response that is typical for this signaling pathway and had lower pSTAT3 expression at increased PRL treatment doses. This is likely due to the lack of Notch1 expression which is thought to have a feedforward loop in the signaling pathway [30–33].

In CRC, STAT3 activation has been shown to upregulate JAG1 expression [20]. When the HT29 cells were treated with 60 ng/mL and 120 ng/mL of prolactin, JAG1 expression significantly increased from baseline levels at the 12-h timepoint (Fig. 2C). Cells treated with 20 ng/mL of PRL did not have a significant increase in JAG1 expression compared to baseline. At this time, the HT29 cells treated with the two higher PRL concentrations showed about a twofold higher JAG1 expression compared to those treated with 20 ng/mL (Fig. 2D). Expression in this group changed dramatically after 12 h of additional incubation with the 20 ng/mL PRL treatment. There was no difference observed when comparing each of the three treatment conditions to each other at the 24-h timepoint. This trend continued until the 48-h timepoint. The COLO320 cells showed an increase in JAG1 expression over time with the same prolactin treatment concentrations, although this was not as high as the changes observed in the HT29 cells (Additional File 2: Fig. S1C and D).

JAG1 is a transmembrane ligand that binds to the Notch1 receptor of neighboring cells, a cellular signaling pathway which is well known to result in increased stemness and EMT markers [34]. The intracellular domain of Notch1 (NICD) is then cleaved and acts downstream as a transcription factor. At all three of the PRL concentrations in the HT29 cells, the NICD increased significantly after 48 h (Fig. 2E). There was no significant difference in NICD expression between the treatment groups at each of the timepoints tested in the HT29 cells (Fig. 2F). Changes in NICD were not investigated for the COLO320 cells as these lack active Notch signaling [30].

### Cancer stem-associated and EMT marker expression increase with prolactin treatments

**Fig. 3:**
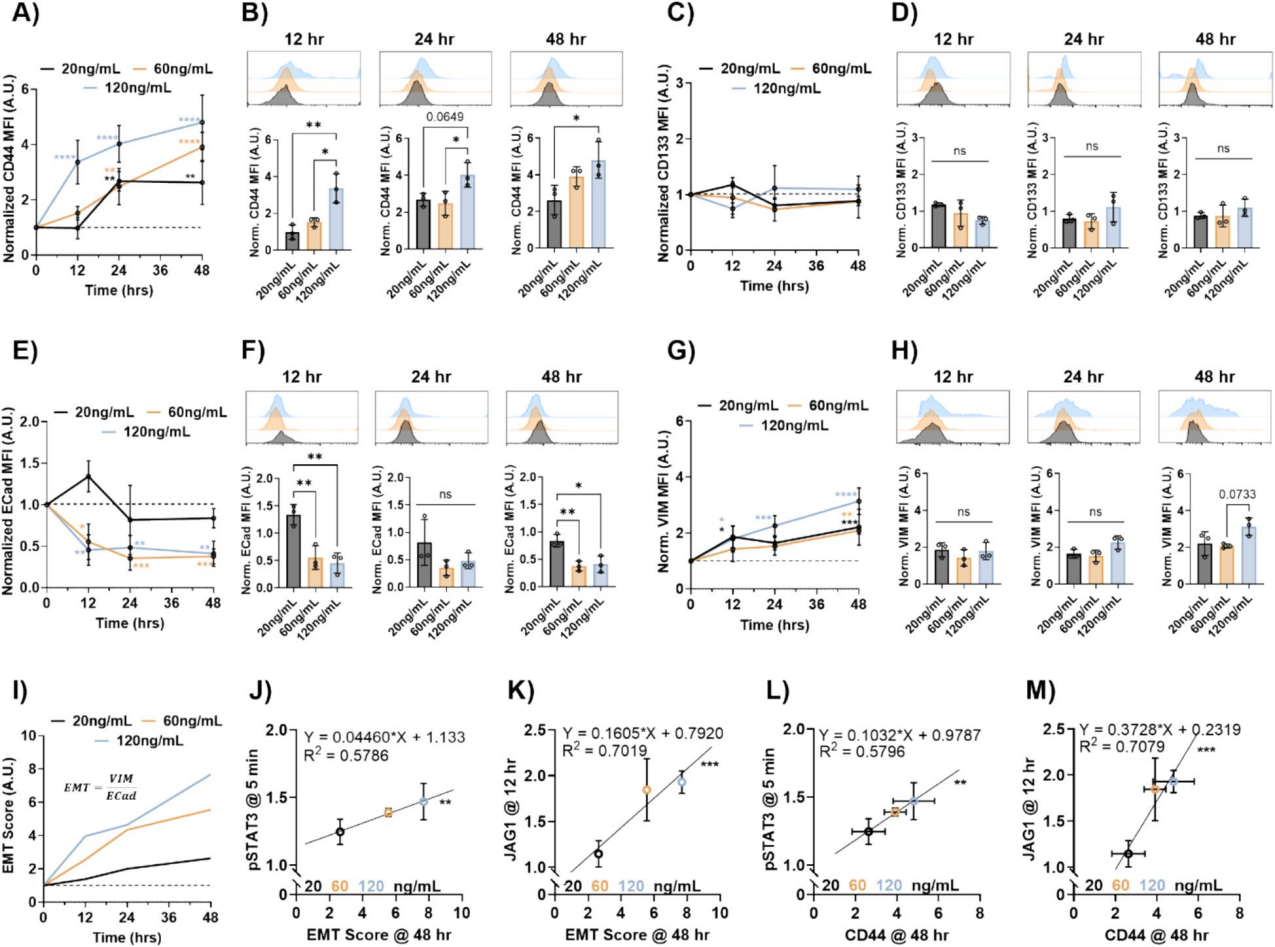
Cancer stem-associated markers and EMT increase with prolactin treatments. **A** XY plot of CD44 expression from 0 to 48 h treated with 20 ng/mL, 60 ng/mL, and 120 ng/mL of PRL. **B** FC histogram and quantification of CD44 expression at 12, 24, and 48 h post-treatment. **C** XY plot of CD133 expression from 0 to 48 h treated with 20 ng/mL, 60 ng/mL, and 120 ng/mL of PRL. **D** FC histogram and quantification of CD133 expression at 12, 24, and 48 h post-treatment. **E** XY plot of E-cadherin expression from 0 to 48 h treated with 20 ng/mL, 60 ng/mL, and 120 ng/mL of PRL. **F** FC histogram and quantification of E-cadherin expression at 12, 24, and 48 h post-treatment. **G** XY plot of vimentin expression from 0 to 48 h treated with 20 ng/mL, 60 ng/mL, and 120 ng/mL of PRL. **H** FC histogram and quantification of vimentin expression at 12, 24, and 48 h post-treatment. **I** EMT score from 0 to 48 h treated with 20 ng/mL, 60 ng/mL, and 120 ng/mL of PRL. Correlation of EMT score with **J** the levels of STAT3 phosphorylation at 5 min and **K** the levels of JAG1 expression at 12 h. Correlation of CD44 expression at 48 h with **L** the levels of STAT3 phosphorylation at 5 min and **M** the levels of JAG1 expression at 12 h. Statistical significance was evaluated by two-way ANOVA or ordinary one-way ANOVA and is shown as *p < 0.05, **p < 0.01, ***p < 0.001, and ****p < 0.0001 (*n* = 3). For the correlation graphs, statistical significance was evaluated by a simple linear regression and is shown as *p <0.05, **p < 0.01, ***p < 0.001, and ****p < 0.0001 for slopes deviating from zero (*n* = 3 biological replicates)

The expressions of CD44 and CD133 were tested to evaluate increases in cancer cell stemness after each of the PRL treatments at 12, 24, and 48 h. At the 12-h timepoint, CD44 expression increased significantly by threefold in the HT29 cells treated with 120 ng/mL of PRL (Fig. 3A). Cells treated with 20 ng/mL and 60 ng/mL of PRL had no significant changes in CD44 expression at 12 h compared to the baseline, and both had significantly lower expression compared to the cells treated with 120 ng/mL (Fig. 3B). CD44 expression continued to rise in the HT29 cells with the highest PRL treatment concentration, reaching five times the expression levels compared to the baseline. Expression in the lower two treatment groups rose slowly in comparison at the 24-h mark. However, this plateaued for the 20 ng/mL treatment group past the 24-h timepoint. At 24 h, the 120 ng/mL treatment group had significantly increased CD44 expression compared to the 60 ng/mL treatment group. At 48 h, the HT29 cells treated with 20 ng/mL had significantly lower CD44 expression in comparison to the 120 ng/mL treated cells. Overall, CD133 expression did not change throughout the experiment with any treatment group compared to the baseline conditions (Fig. 3C). No difference in CD133 mean fluorescence intensity (MFI) was found between any of the treatment concentrations at 12, 24, or 48 h (Fig. 3D). Interestingly, the HT29 cells treated with 120 ng/mL of PRL showed the emergence of a CD133+ subpopulation in the flow cytometry plots starting at the 24-h timepoint, a marker which is linked to stem cell-like properties. No significant changes in CD44 or CD133 were observed in the COLO320 cells after prolactin treatment (Additional File 2: Fig. S2A, B, C, D). This was expected since they are downstream of Notch1 activity in the pathway.

Vimentin and E-cadherin expression was evaluated to confirm that pregnancy PRL levels induce EMT in CRC cells. When the HT29 cells were treated with 20 ng/mL of PRL, there was no significant change in E-cadherin expression at any of the three timepoints tested (Fig. 3E). In contrast, E-cadherin expression decreased on average by 50% in the HT29 cells treated with 60 ng/mL and 120 ng/mL at the 12-h timepoint compared to the baseline. The HT29 cells treated with 20 ng/mL had significantly higher E-cadherin expression than those treated with 60 ng/mL and 120 ng/mL (Fig. 3F). Significantly lower E-cadherin expression was sustained out to the 24-h and 48-h timepoints for the HT29 cells treated with the two higher PRL concentrations compared to the baseline values. Again, HT29 cells treated with 20 ng/mL had significantly more E-cadherin than those treated with 60 ng/mL and 120 ng/mL. As expected, the opposite trend was observed in vimentin expression. HT29 cells in all three treatment groups had steadily increased vimentin expression with time (Fig. 3G). The cells treated with 120 ng/mL had significantly higher vimentin expression compared to the baseline at 12, 24, and 48 h. At the 48-h timepoint, the vimentin expression after treatment with 20 ng/mL and 60 ng/mL was significantly increased compared to the baseline values. Comparing the three treatment groups at each timepoint, there were no significant differences in vimentin expression between the higher, intermediate, and lower doses (Fig. 3H), although there was a trend showing higher vimentin expression in the cells treated with 120 ng/mL compared to the other two doses. No significant changes in vimentin or E-cadherin were observed in the COLO320 cells after prolactin treatment (Additional File 2: Fig. S2E, F, G, H). This was expected since they are downstream of Notch1 activity in the pathway.

To further explore the extent of EMT, the EMT score was quantified at each timepoint for each of the three PRL treatment concentrations used in the HT29 cells. This was calculated by dividing the mean vimentin expression by the mean E-cadherin expression for each dose at each timepoint [35]. The calculated EMT score indicated that PRL had the strongest effect at the concentration found in late pregnancy (120 ng/mL) (Fig. 3I). HT29 cells treated with 20 ng/mL scored lowest for EMT but still increased steadily over time. HT29 cells treated with 60 ng/mL fell between the other two treatments at all time points; however, the EMT score was closer to that of the higher dose. This indicated that the extent of EMT is tied to pregnancy-associated PRL concentrations. The different EMT score endpoints at the 48-h timepoint were plotted against upstream protein expression at distinct time points. These are the timepoints where the protein expression or activation was found to vary significantly with changes in PRL dosage. For pSTAT3, the greatest dose-dependance was observed at the 5-min timepoint. The extent of fast STAT3 phosphorylation correlated positively with EMT score, and the slope was shown to deviate significantly from zero (Fig. 3J). Dose-dependance was the greatest for JAG1 at the 12-h timepoint. When plotted together, JAG1 expression correlated with EMT score, and the slope was shown to deviate significantly from zero (Fig. 3K). Interestingly, these trends were also observed when plotting STAT3 phosphorylation at 5 min and JAG1 at 12 h with the endpoint CD44 expression. Both linear regressions show that these were positively correlated, and the slopes were significantly different from zero (Fig. 3L and M).

### Computational model of the JAK2/STAT3 signaling pathway in response to late-pregnancy PRL levels

**Fig. 4:**
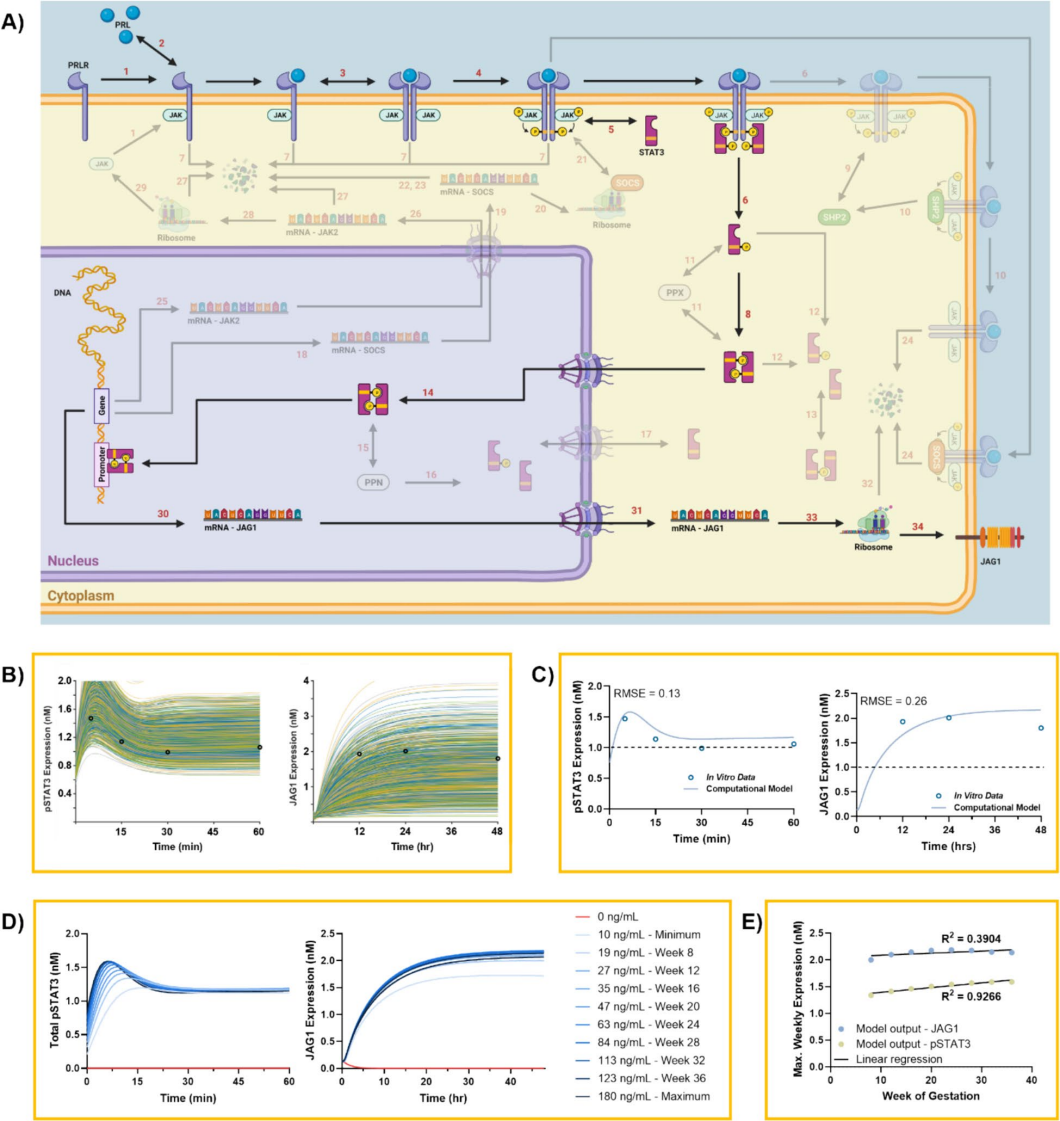
Computational model of the JAK2/STAT3 signaling pathway in response to late-pregnancy PRL levels. A Schematic detailing the activation of the JAK2/STAT3 pathway resulting in increased JAG1 expression on the cell membrane. The reactions that make up the computational model are exactly labeled to match each of the calculations performed in the mechanism file as to make the model code and the MATLAB data output tables easier to navigate with this visual aid. **B** Plots of 1000 random iterations of the pSTAT3 and JAG1 concentration curves from the second set of Monte Carlo simulations. **C** Plots of the best fit curves for pSTAT3 and JAG1 expression to the *in vitro* data, corresponding to iteration number 21455. **D** Fitted model outputs for pSTAT3 and JAG1 using input mean PRL values from every 4 weeks throughout gestation, as well as total minimum and maximum recorded levels from *Biswas et al. (1976)*. **E** Maximum expression of pSTAT3 and JAG1 using input mean PRL values from every 4 weeks throughout gestation

A computational model was built to further investigate the activation of the STAT3/JAK2/JAG1 pathway in response to late-pregnancy levels of PRL. The model follows the canonical JAK/STAT activation pathway outlined in Fig. 4A, starting with the binding of JAK2 (R1) to the intracellular domain of the PRLR. PRL then binds to the PRLR-JAK complex (R2–4) and initiates the activation of the pathway which proceeds in the cytoplasm of the cell. Inactive STAT3 binds to the PRL-PRLR-JAK2 cluster (R5), is phosphorylated (pSTAT3) (R6), and then forms a dimer in the cytoplasm (R8). The dimer passes through a nuclear pore to bind with the DNA (R14) and acts as a transcription factor for JAG1. Lastly, JAG1 mRNA is transcribed, exits the nucleus, is translated into a protein, and is expressed on the cell membrane (R30-34). There are three integrated regulatory modules that control JAK/STAT phosphorylation: dephosphorylation by the PPX/PPN (R11–17), the SOCS, (R18–24), and the SHP2 modules (R9 & 10).

The initial fit of the computational model using the protein expression and kinetic constant values from the *Mortlock *et al*. (2020)* model (found in Tables S1 & S2) to the in vitro data for STAT3 phosphorylation can be found in Additional File 2: Fig. S3A. Data from the computational model replicated the characteristic curve for STAT3 phosphorylation, yet the magnitude of phosphorylation calculated in response to 120 ng/mL of PRL was, on average, double that of the in vitro data. Overall, this initial fit of the model STAT3 phosphorylation to the in vitro data was poor, with a root-mean-square error (RMSE) of 0.79. The computational model also showed a poor fit to the JAG1 experimental results, with an RMSE of 0.84 (Additional File 2: Fig. S3B). This is likely due to the difference in the endpoint proteins of interest in the two different models and the difference in cell types and species. These values were nevertheless useful as a starting point for finding the initial conditions and parameters that would achieve a better fit.

The computational model was fit to all of the in vitro pSTAT3 and JAG1 expression values simultaneously via a two-step Monte Carlo simulation. Initial conditions were varied randomly with a normal distribution and were calculated based on the fitted values from the *Mortlock *et al*. (2020)* model [36]. For this first step of the model fitting, 10,000 iterations were run while randomly varying the initial conditions and kinetic parameters by a maximum of ± 100% of the initially guessed values with a Gaussian distribution. For visualization, 1000 random simulation outputs for pSTAT3 were plotted over time where each line in the graph is an individual iteration (Additional File 2: Fig. S4A). Most iterations reached a maximum point of STAT3 phosphorylation between 5 and 15 min, similar to the timescales for protein phosphorylation in cells. The majority of the iterations did not return to baseline values at or before 60 min. All of the simulation outputs for JAG1 were also plotted over time. In the case of JAG1, there was a much larger variation than with pSTAT3 as it is further downstream. The coarse model fit was improved by utilizing the optimized parameter set for initial conditions and kinetic constants from iteration 7418 of the first Monte Carlo simulation (Additional File 2: Fig. S4B). Here, the RMSE for pSTAT3 was reduced by 80%, compared to the fit with the initial guesses, to 0.16. The RMSE for JAG1 was calculated to be 0.62, having only decreased by 26%. The values for the fitted parameters can be found in Table S3. Parameters that had positive change between the initial values and the fitted values are shown in blue, and those with negative change are shown in red. Histograms of the distribution of each of the randomly varied kinetic constants and initial protein concentrations can be found in Additional File 2: Fig. S4C and D and Additional File 2: Fig. S5.

To get an improved fit for the model outputs to the in vitro results, a second Monte Carlo simulation was run. The initial conditions and kinetic parameters were varied randomly with a Gaussian distribution for a maximum of ± 30% of the values from iteration 7418 of the first Monte Carlo simulation. In this case, 30,000 iterations of the model were run. For visualization, 1000 random simulation outputs for pSTAT3 and JAG1 were plotted over time, where each line in the graph is an individual iteration (Fig. 4B). The iteration of the second Monte Carlo simulation with the best fit was number 21455. The output lines for pSTAT3 and JAG1 for this iteration were plotted individually, and the RMSE for each was calculated (Fig. 4C). Here, the RMSE for pSTAT3 was reduced to 0.13. This was a small decrease of 19% compared to the best-fit iteration from the first Monte Carlo simulation. Overall, the pSTAT3 RMSE decreased by 84% compared to the model output using the initial guesses. The RMSE for JAG1 was calculated to be 0.26. Compared to the best-fitting iteration from the first Monte Carlo simulation, it decreased by 58%. The JAG1 RMSE decreased by 70% compared to the model output using the initial guesses. The values for the fitted parameters can be seen in Table S3. Overall, this suggests the successful regression of the computational model for PRL activation of the JAK/STAT pathway leading to increased JAG1 expression, as seen in the literature and the previous in vitro results.

To assess the robustness of our fitted parameters, we performed three additional Monte Carlo simulations centered on the best‐fit values, allowing each kinetic constant and initial protein concentration to vary within ± 100%, ± 50%, ± 10%, or ± 2% of its optimized value. With a 100% and 50% perturbation of the initial parameter values, a large variation occurred in both the pSTAT3 and JAG1 curves (Additional File 2: Fig. S6A). This was markedly reduced once the tolerance was reduced to 10% and further with 2%. Specifically, when there was a random 2% change in parameter values, the maximum pSTAT3 and JAG1 values were minimally impacted compared to the fitted model outputs (Additional File 2: Fig. S6B and C). The RMSE between each ensemble and the baseline was smallest for the 2% perturbation, confirming that modest parameter deviations have minimal impact on model outputs.

The best-fit model was then used to further investigate the signaling pathway and to simulate pSTAT3 and JAG1 responses across the physiological range of pregnancy-associated PRL levels [10]. The PRL serum concentrations for weeks 8 (19 ng/mL), 12 (27 ng/mL), 16 (35 ng/mL), 20 (47 ng/mL), 24 (63 ng/mL), 28 (84 ng/mL), 32 (113 ng/mL), and 36 (123 ng/mL) during pregnancy were input into the model in addition to a 0 ng/mL control, and the minimum (10 ng/mL) and maximum (180 ng/mL) serum PRL levels were recorded. The initial PRL concentration strongly affected pSTAT3 curve magnitude and timing within the computational model (Fig. 4D). In contrast, the effects of increasing PRL on JAG1 expression were more modest and plateaued by week 32. This matched the observations made in the in vitro experiments. As expected, the 0 ng/mL PRL control produced no additional pSTAT3 or JAG1 confirming that prolactin input is essential for pathway activation. The maximum from the pSTA3 and JAG curves was plotted against the gestational week (Fig. 4E). This revealed that pSTAT3 peak magnitude correlates strongly with advancing pregnancy (*R*^2^ = 0.93), whereas JAG1 expression showed a lower dependance.

The fitted model was then subjected to a sensitivity analysis where it was run through 49 iterations changing the kinetic constants individually to 0. The output curves for pSTAT3 and JAG1 in response to these changes can be observed in Additional File 2: Fig. S7A and D. The sensitivity analysis allows us to use RMSE values to identify which reactions have the greatest impact on signaling dynamics within the model. A scatter plot of the RMSE values of pSTAT vs JAG1 show that k14 (movement of dimerized pSTAT3 into the nucleus), k15r (PPN-mediated dephosphorylation), k30a (mRNA transcription), k31 (mRNA exiting the nucleus), k33 (protein translation), and deg_ratio (protein degradation with time) have the largest roles within the model (Additional File 2: Fig. S7C). With regard to STAT3 phosphorylation, the largest RMSE values were observed for reactions in the first half of the signaling pathway during PRL-receptor binding, dimerization, and activation events (Additional File 2: Fig. S7D). Additionally, feedback regulators, especially those linked to the receptor complex and SOCS, play a large role in the magnitude and timing of STAT3 phosphorylation. The JAG1 expression was more sensitive to changes throughout the signaling pathway as it is found at the end of the signaling pathway (Additional File 2: Fig. S7E). The RMSE values were largest for reactions governing JAG1 mRNA transcription (k30a), protein translation (k33), and nuclear translocation (k14 and k31).

#### Prolactin sensitizes HT29 cells to TRAIL-mediated apoptosis

**Fig. 5:**
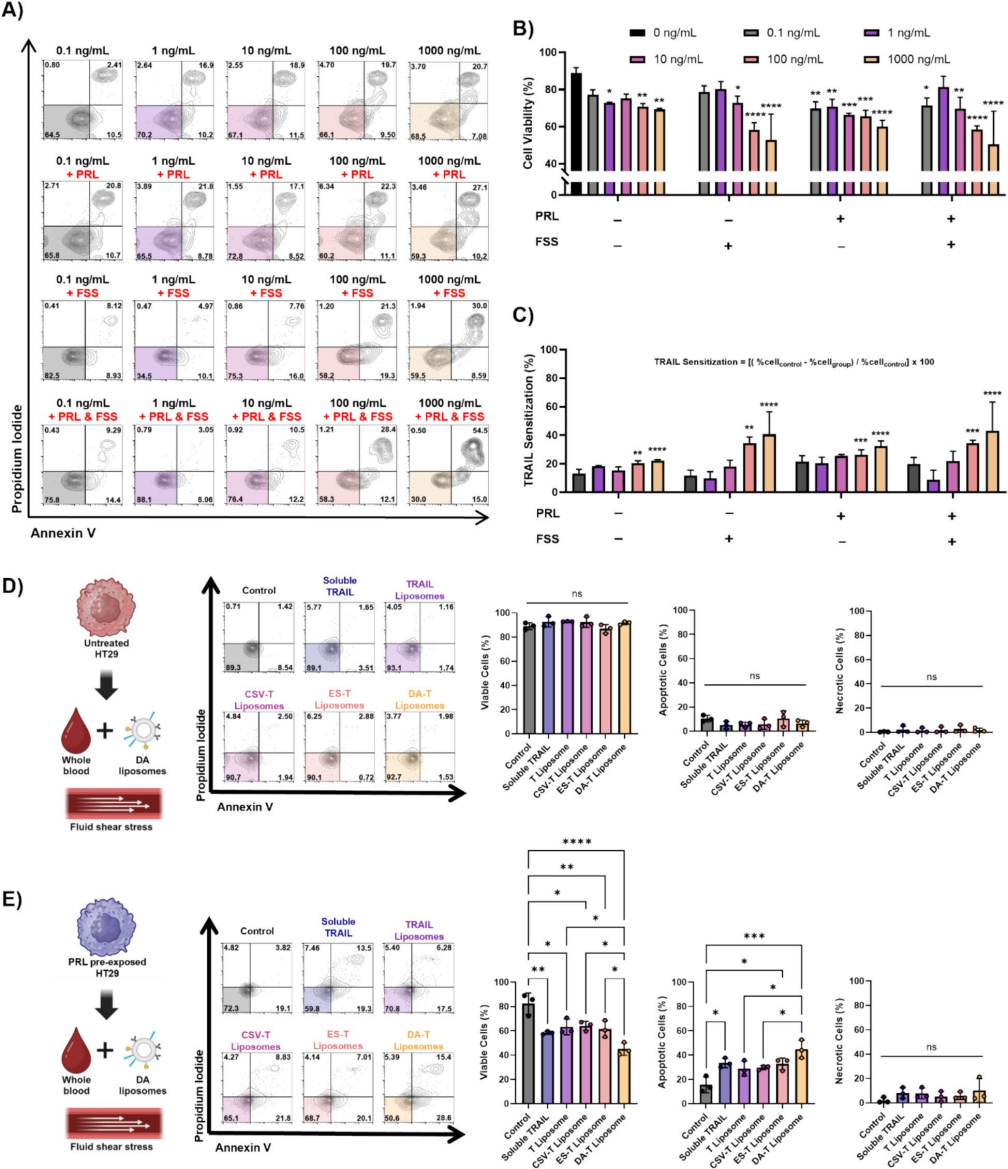
Prolactin sensitizes HT29 cells to TRAIL-mediated apoptosis. **A** Representative flow cytometry plots of the HT29 cells treated with 0.1 to 1000 ng/mL of soluble TRAIL in static conditions, under FSS, after PRL pre-exposure, and in FSS combined with PRL pre-exposure. **B** Quantification of the cell viability after soluble TRAIL treatment (statistical analysis was performed comparing all groups to the static 0 ng/mL TRAIL dosage without PRL or FSS). **C** Quantification of the TRAIL sensitization by each of the experimental groups (statistical analysis was performed comparing all groups to the 0.1 ng/mL TRAIL group without PRL or FSS). **D** Representative flow cytometry plot and quantification of cell viability, apoptosis, and necrosis of the HT29 cells treated with the DA liposomes in whole blood under FSS conditions. **E** Representative flow cytometry plot and quantification of cell viability, apoptosis, and necrosis of the PRL pre-exposed HT29 cells treated with the DA liposomes in whole blood under FSS conditions. Statistical significance was evaluated by either an ordinary one-way ANOVA or a two-way ANOVA and is shown as *p < 0.05, **p < 0.01, ***p < 0.001, and ****p < 0.0001 (*n* = 3 biological replicates)

First, the effect of TRAIL treatment on colorectal cancer cells that had been previously exposed to pregnancy levels of PRL was evaluated under static and shear conditions (Fig. 5A). Performing the experiments under shear conditions is important as TRAIL is most effective under fluid shear stress (FSS), making it an attractive treatment strategy for disease progression prevention in pregnant CRC patients who have been shown to have a high metastatic burden [1]. When HT29 cells were treated with soluble TRAIL concentrations ranging from 0.1 to 1000 ng/mL under static conditions, cell viability decreased in the high concentration treatment groups (Fig. 5B). This indicates that without PRL pre-exposure or external mechanical forces, these cells are resistant to TRAIL-mediated apoptosis at low dosages, as seen in the literature [37]. HT29 cells were then exposed to late-pregnancy levels of PRL (120 ng/mL) for 3 days and then treated with TRAIL for 24 h. Exposure of non-treated HT29 cells to TRAIL under FSS conditions only significantly increased cell death at high TRAIL dosages. In comparison, all PRL-exposed cells treated with TRAIL had significantly lower cell viability after 24 h compared to the control group. Similar results were observed when HT29 cells were exposed to PRL and treated with TRAIL under FSS conditions. The extent of TRAIL sensitization in each group was calculated by subtracting the cell viability of the tested group with the cell viability of the control group (0 ng/mL TRAIL in static conditions) divided by the cell viability of the control group (Eq. 5). Pre-exposing HT29 cells to pregnancy prolactin levels significantly increased sensitization to TRAIL-mediated apoptosis when using high TRAIL concentrations (Fig. 5C). This trend was also observed in the groups treated under FSS conditions and the combination treatments.

Membrane-bound TRAIL is known to have higher efficacy in inducing apoptosis in cancer cells compared to soluble TRAIL [38]. Since 2014, the King Lab has developed various liposome designs, decorated with TRAIL, focused on killing CTCs in the bloodstream by tethering loosely to healthy leukocytes [25]. A recent iteration of these liposomes was designed to transfer from the surface of the leukocytes and bind to the surface of CTCs, thereby increasing the circulation time of the liposomes but also adding a targeted approach to the drug delivery system [26]. The surface of the dual affinity (DA) liposomes is functionalized with TRAIL, anti-cell surface vimentin (CSV) antibody fragments to target the CTC, and E-selectin (ES) protein to reversibly bind to healthy leukocytes. To further test the use of TRAIL as a therapeutic for colorectal cancers during pregnancy, HT29 cells were treated with these dual affinity liposomes under FSS in whole blood to better simulate the environment found in the circulation. Several different formulations of liposomes were used for comparison: TRAIL-only liposomes, anti-CSV/TRAIL liposomes, and ES/TRAIL liposomes. A no-treatment control and soluble TRAIL control were also used. No significant changes in cell viability were observed when the HT29 cells were exposed to the DA liposomes under FSS conditions (Fig. 5D). In comparison, when the cells were preexposed to pregnancy levels of PRL (120 ng/mL) for 3 days before treatment with the TRAIL-coated liposomes under the same conditions, there was a significant increase in cell death (Fig. 5E). The treatment efficacy is especially pronounced in the presence of the DA liposomes, where there was a decreased cell viability of 45% in the prolactin-exposed cells compared to those that did not receive any treatment. This shows that TRAIL-resistant HT29 cells can be successfully killed using TRAIL under FSS conditions of the circulation, in particular when the cells have been previously exposed to pregnancy levels of PRL and when TRAIL is in liposomal form.

## Discussion

Cancer during pregnancy is a rare occurrence, yet it presents added complications in a vulnerable patient population. Specifically, if CRC is diagnosed during pregnancy, it can lead to more aggressive disease presentation. The analysis of the pregnant CRC patient population at Vanderbilt University Medical Center showed that the majority of tumors were located in the rectum, correlating with early-onset colorectal cancer (EOCRC) which largely presents with left sided colon and rectal tumors [39]. There are three compounding factors which lead to this phenomenon (Fig. 1). The third factor is based on evidence from the literature that prolactin can enhance CRC malignancy [18]. In this study, the link between increased levels of PRL observed during pregnancy, overactivation of the PRL/STAT3/JAG1 pathway in CRC, and increased EMT and cancer cell stemness was established by in vitro and in silico experimentation. CRC cells treated with late-pregnancy levels of PRL showed significantly increased STAT3 phosphorylation and JAG1 expression (Fig. 2). An increase in EMT and CD44, the cancer stem cell marker, with pregnancy levels of PRL dose, was also confirmed (Fig. 3). This further illustrates why this small yet high-risk patient group seems to show increased disease aggressiveness and metastatic progression compared to non-pregnant patients with early-onset CRC.

Previous studies in the literature used the CRC cell line HCT116 to investigate the effects of PRL, yet these cells were isolated from a male patient [18]. Research on woman-specific biology has risen recently but is still scarce and poorly understood because scientific advances have been primarily focused on male biology in basic and preclinical studies [40]. Specifically, the effects of pregnancy on the prevention, management, and treatment of disease are critically understudied [41]. Therefore, it is important to utilize a cancer cell line isolated from female patients when possible, especially when focusing on women-centric cancer comorbidities. On the ATCC website, there are a total of 52 human colorectal cell lines available for cancer research applications. Of these, only six are derived from female patients, highlighting that there is a disproportionate focus on male disease even at the level of research material availability. HT29 was chosen as the model cell line for the in vitro experimentation as they are the most common CRC cell line isolated from a female patient utilized in the literature and is also Notch1 positive [42, 43]. The COLO320 line was chosen as it is also one of the few available colorectal cancer cell lines via ATCC that is derived from a female patient and positive for the prolactin receptor, allowing us to explore the phosphorylation of STAT3 in response to prolactin treatment. It was particularly interesting to observe the lack of changes in downstream EMT and stemness markers due to the Notch1 deficiency, highlighting the importance of this protein in the signaling pathway.

The computational model was adapted from the *Mortlock *et al*. (2020)* study and fit using a two-step Monte Carlo simulation [36]. The model from the literature that was used as a base for our model was built to understand the JAK2/STAT5A&B activation due to PRL in rat pancreatic beta cells resulting in increased Bcl-2 protein expression. Our model was successfully adapted by exchanging the STAT5-related proteins to STAT3. Additionally, Bcl-2 expression calculations were replaced for JAG1. Therefore, to fit this model to the in vitro data generated from female-derived prolactin-treated CRC cells, two Monte Carlo simulations were run in series to first determine the coarse fit of the model and then generate a finer fit using 10,000 and 30,000 iterations, respectively (Fig. 4). Interestingly, the greatest change between the original and fitted kinetic constants was observed in the kinetic constants which govern JAG1 expression in the model. This is likely due to the fact that JAG1 expression varies at a different rate and magnitude compared to Bcl-2 and therefore required the greatest change in parameters. Additionally, since JAG1 expression is one of the last to be calculated in the computational model, the effect of upstream changes in parameters has a greater influence than on pSTAT3 which is the 11th protein in the signaling cascade.

Lastly, we utilized the previously developed dual affinity TRAIL-liposome system to investigate the efficacy of this safer therapy option [26]. While the results for PRL sensitization of HT29 cells are exciting, further work needs to be done to better understand the underlying mechanisms of this effect. When colorectal cancer cells that had previously been exposed to pregnancy levels of prolactin were treated with the dual affinity liposomes in whole blood under physiologically relevant shear stress, there was a significant decrease in cell viability (Fig. 5). While these results are promising, more work needs to be done to confirm that liposomal TRAIL can provide a more effective and safe treatment option against metastasizing cancer cells that have been exposed to pregnancy levels of prolactin.

## Conclusions

Overall, a correlation was established between the high late-pregnancy levels of PRL and increased cancer EMT and stemness via in vitro and in silico experimentation. The female CRC cell line HT29 was treated with three different concentrations of PRL and confirmed to exhibit dose- and time-dependent increases in pSTAT3, JAG1, CD44, and EMT score. A computational model was then built to calculate changes in the JAK2/STAT3/JAG1 in response to PRL treatments and successfully fit to the in vitro data. This allows us to further investigate the link at a cellular level between the increased cancer aggressiveness observed in pregnant CRC patients and the activation of EMT and stemness pathways due to high PRL levels in the third trimester. The computational model and presented results can help guide mechanistic follow-up experiments and the selection of potential therapeutic targets. Additionally, we suggest an alternative targeted treatment option to help reduce the prevalence of circulating tumor cells in CRC patients who are pregnant.

## Methods

### CRC-pregnancy patient data collection

This protocol involving human subjects was approved by the Institutional Review Board at Vanderbilt University, and informed consent was obtained from all patients whose information was collected prospectively (IRB no. 220852 “Data and Sample Collection Pregnant and Non-Pregnant Colorectal Cancer Patients”). Those patients whose information was collected retrospectively was done without the collection of protected health information (PHI). All pregnant women that were histologically or cytologically confirmed EOCRC being treated at Vanderbilt University Medical Center were considered for this study. The control group of patients was chosen based on matching the age of the pregnant CRC patients and the cancer stage to allow for direct comparison of tumor size, treatment options, and disease outcomes. For the age-/stage-matched comparison, women who are not pregnant and who were histologically or cytologically confirmed early-onset colorectal cancer being treated at Vanderbilt University Medical Center were considered for this study. EOCRC was defined as those diagnosed under 45; therefore, for the purpose of this study, only women between the ages of 25–45 years old were considered. All other diagnoses were excluded. Right-sided tumors included the appendix, the cecum, the ascending colon, the hepatic flexure, and the transverse colon. Left-sided tumors included the splenic flexure, the descending colon, and the sigmoid colon.

The clinicopathologic data of each patient was collected by key study personnel both retrospectively and prospectively. The information collected included the following: Patient demographic data, medical history, cancer diagnosis, diagnostic evaluations, cancer treatment history, disease status, laboratory values including molecular profiling, radiographic studies, pathology reports, surgery reports, radiation reports, drug administration records, and clinical outcomes (response to treatment, side effects, progression or relapse-free survival, overall survival, etc.). This project utilized Vanderbilt University Medical Center’s REDCap electronic data capture platform for data collection and management.

### Cell culture and reagents

The human colon adenocarcinoma cell lines HT29 (HTB38, ATCC) and COLO320DM (CCL-220, ATCC) were used throughout the study. These cell lines were chosen because they were both isolated from female patients, an important consideration since this study is focused on a women’s health issue. The HT29 cell line was isolated from a colorectal adenocarcinoma primary tumor obtained from a 44-year-old, white, female patient in 1964. It has an epithelial morphology and is positive for Notch1. The COLO320DM was isolated from a colorectal adenocarcinoma tumor (Duke’s type C) obtained from a 55-year-old, white, female patient. It is a rounded and refractile cell line and Notch1 deficient. Due to the severe underrepresentation of samples isolated from female patients in commercial cell banks, described in further detail in the “Discussion” section, variability in race and age of the patients from which the cell lines were isolated was not explored. The HT29 cells were cultured in McCoy’s media (Gibco, 16,600–082) supplemented with 10% (v/v) FBS (Gibco, 26,140–079) and 1% (v/v) penicillin–streptomycin solution (Corning, 30002CI). The COLO320DM cells were cultured in RPMI-1640 media (Gibco, 11,875–093) supplemented with 10% (v/v) FBS and 1% (v/v) penicillin–streptomycin solution. Standard culture conditions were maintained. Before treatments began, 200,000 cells were plated in a 48-well plate and cultured for 48 h. The treatment times tested for STAT3 phosphorylation were the following: 5 min, 15 min, 30 min, 1 h, 3 h, and 12 h. The treatment times tested for all other proteins were 12, 24, and 48 h. The human PRL (Thermo Fisher Scientific, 100–07-250UG) concentrations used were 20 ng/mL, 60 ng/mL, and 120 ng/mL. A group without PRL treatment was used to evaluate the baseline protein expressions at *t* = 0.

### Flow cytometry

Once the timepoints were reached, cells were detached via trypsinization, fixed in 4% (v/v) PFA (15,714-S, Electron Microscopy Sciences) in HBSS for 15 min, and then permeabilized in 1% (v/v) Triton X-100 (9002–93-1, Sigma Aldrich) in HBSS (990 µL) for 10 min. A 30-min blocking step in 5% BSA (A1470-100G, Sigma Aldrich) in HBSS was then performed. Cells were incubated with antibodies for pSTAT3 (11–9033-42, Thermo Scientific), JAG1 (sc-390177, Santa Cruz Biotechnology), NICD (4147S, Cell Signaling Technology), CD44 (555,478, BD Biosciences), CD133 (293C3, MACS Miltenyi Biotech), vimentin (V6630, Sigma Aldrich), and E-cadherin (sc-67A4, Santa Cruz Biotechnology) overnight at 4 °C at a ratio of 1:100 in 5% BSA in HBSS and washed in HBSS with Ca^2+^ and Mg^2+^. For the vimentin stain, samples were incubated with the AF555 rabbit antihuman IgG (H + L) secondary antibody (A-21428, Invitrogen) in 5% BSA in HBSS at a ratio of 1:250 for 30 min at RT. For the NICD stain, samples were incubated with the AF488 mouse antihuman IgG (H + L) secondary antibody (A28175, Thermo Scientific) in 5% BSA in HBSS at a ratio of 1:250 for 30 min at RT. Again, the cells were washed in HBSS with Ca^2+^ and Mg^2+^. All other antibodies were pre-conjugated.

Samples were run through a Guava EasyCyte 12HT Flow Cytometer using the Green-B, Yellow-B, and Red-R lasers. Single stain controls were used for compensation, and unstained controls were used to measure background fluorescence for noise removal in normalization. Flow cytometry data was analyzed on FlowJo. Normalized protein expression was calculated using Eq. 1. Here, *F*_Protein_ is the mean fluorescence intensity (MFI) of the protein at any timepoint, *F*_Baseline_ is the MFI of the protein at *t* = 0 with no treatment, and *F*_Background_ is the MFI of the unstained control for background noise. The EMT score was calculated using Eq. 2. Here, we divide the mean of each of the replicates of the normalized vimentin expression (*VIM Expression*_*Normalized*_) at each time point by the mean of each of the replicates of the normalized E-cadherin expression (*Ecad Expression*_Normalized_) at each timepoint. Data are available in Additional File 2: Data S1 and S2.

1 $${Protein expression}_{Normalized}=\frac{{F}_{Protein}-{F}_{Background}}{{F}_{Baseline}-{F}_{Background}}$$

2 $$EMT score= \frac{Mean({VIM Expression}_{Normalized)}}{Mean({ECad Expression}_{Normalized)}}$$

### Computational model of the PRL/STAT3/JAG1 pathway

A mathematical model was built to detail the JAK2/STAT3 activation pathway in response to pregnancy levels of PRL resulting in upregulated JAG1 expression. The model was adapted by defining a system of ordinary differential equations (ODEs) based on the reaction steps and kinetic values outlined in the JAK/STAT signaling models by *Mortlock *et al*. (2020)* and *Yamada *et al*. (2003)* [36, 44]. Specifically, the *Mortlock *et al*. (2020)* model provides useful details related to the reaction kinetics of the JAK2/STAT5A&B in rat pancreatic beta cells in response to PRL binding [36]. The chemical reaction network is defined by mass action kinetics, and each ODE defines the time-dependent changes in concentration of each species according to the kinetic constant and whether it is a reactant or product in a given reaction [45]. Initial values for protein expression and kinetic constants were taken from the *Mortlock *et al*. (2020)* model, mixing values in the literature with fitted values from their Monte Carlo simulation (Supplementary Tables 1 and 2). The initial values for PRL treatment concentrations were the same as the in vitro experiments: 20 ng/mL, 60 ng/mL, and 120 ng/mL. The simulation was run from 0 to 48 h in 60 s increments. MATLAB was used to perform the model calculations. The MATLAB code and computational data files can be found on Zenodo (10.5281/zenodo.16639924) [46].

### Computational model parameter fitting via Monte Carlo simulations

The computational model was fitted to all the in vitro data simultaneously to perform the parameter fitting. To do so, two Monte Carlo simulations were run in series to determine the distribution of STAT3 phosphorylation and JAG1 expression. Parameters and initial conditions were varied randomly (*P*_rand_) in each iteration with a Gaussian distribution using Eq. 3. The parameters chosen to be varied were those that were found to govern STAT phosphorylation the most in the model analysis by *Mortlock *et al*. (2020)*. These were RJ, PPX, S3c, and S3n for the proteins. The kinetic constants governing PRL-receptor dynamics (k2f, k2r, k4, k5f, and k6), protein degradation (deg_ratio), STAT phosphorylation (k8f), regulatory modules (k11f, k12, and k23), RNA transcription (k25_1 and k30_1), and expression of JAG1 (k28, k31, k33, and k34) were varied. Independent matrices were created for each protein and kinetic constant with 10,000 randomly generated numbers between 0 and 1 in a normal distribution (*RAND*). The random values were multiplied by the initial parameter value (*P*_init_) and a variation constant (*C*_max_). Protein expressions and kinetic constants were varied by *C*_max_. Values were then divided by 3 as 99.7% of the normal curve fell between − 3 and 3 and then subtracted from the original parameter value (*P*_init_). Lastly, the absolute value was taken to prevent any negative protein expression or kinetic constant values.

The first Monte Carlo simulation was run to find a coarse fit of the initial parameters to the experimental data. A total of 10,000 iterations of the simulation were run, varying the initial conditions and parameters by ± 100% (*C*_max_ = 1). Histograms of initial conditions and parameter variations for the first Monte Carlo simulation can be found in Additional File 2: Fig. S4. Once all the iterations of the Monte Carlo simulation had run, the root-mean-square error (RMSE) for the JAG1 expression and STAT3 phosphorylation was calculated to identify the parameter set with the best fit using Eq. 4. These were compared to the pSTAT3 model outputs for each iteration at 5 min, 15 min, 30 min, and 60 min. For JAG1 expression, the RMSE was calculated using the model outputs for each iteration at 12, 24, and 48 h.

3 $${P}_{rand}=\left|{P}_{init}-\left({{C}_{max}\times P}_{init}\times RAND\times \frac{1}{3}\right)\right|$$

4 $$RMSE=\sqrt{ \frac{1}{n} \sum_{i=1}^{n}{({Model\_Data}_{i}-{InVitro\_Data}_{i})}^{2}}$$

The iteration with the outputs that best fit the experimental data was calculated to minimize the RMSE for pSTAT3 and JAG1. The iteration where the point (*RMSE*_pSTAT3_, *RMSE*_JAG1_) had the shortest distance from the point (0,0) was chosen as the best fit. A second Monte Carlo simulation was then executed to obtain the fine fit of the model to the experimental data. The initial conditions and parameter set from the best-fit iteration of the first Monte Carlo simulation were used as the starting point. A total of 30,000 iterations of the simulation were run, varying the initial conditions and parameters by ± 30% (*C*_max_ = 0.3). Histograms of initial conditions and parameter variations for the first Monte Carlo simulation can be found in Additional File 2: Fig. S5. The best-fit iteration was calculated as described above. Lastly, using the fitted kinetic constant and initial protein concentration values, a series of smaller scale Monte Carlo simulations were run to find a *C*_max_ value to provide a range of suitable parameter values where the model would still be relevant. For this, *C*_max_ was set to 100%, 50%, 10%, and 2%, and the model was run for 10,000 iterations.

Once the model was fit to the optimal parameter sets, 10 iterations of it were run using mean PRL input values that from each week of gestation as reported by *Biswas *et al*. (1976)*. These were as follows: 10 ng/mL (total minimum), 19 ng/mL (week 8), 27 ng/mL (week 12), 35 ng/mL (week 16), 47 ng/mL (week 20), 63 ng/mL (week 24), 84 ng/mL (week 28), 113 ng/mL (week 32), 123 ng/mL (week 36), and 180 ng/mL (total maximum).

### Computational model sensitivity analysis

A baseline simulation was first run using the fitted parameter set to obtain reference curves for pSTAT3 and JAG1. Each iteration was run using 120 ng/mL PRL. A parameter perturbation was then performed in 49 iterations of the model where exactly 1 parameter was set to 0 (or 10^−6^ in the case of denominators) while holding all others at the baseline value. For each run, the RMSE was calculated using Eq. 4 comparing the outputs for pSTAT3 and JAG1 from the perturbed iteration to the fitted values of the model.

### TRAIL sensitization assay

In preparation, 200,000 HT29 cells were plated in a 24-well plate. After 24 h, cells were treated with 120 ng/mL of PRL and incubated for 4 days. The cells were then treated with 0.1, 1, 10, 100, and 1000 ng/mL of recombinant human soluble TRAIL (310–04, PeproTech) reconstituted in HBSS with Ca^2+^ and Mg^2+^. A 0.1 ng/mL dose of TRAIL was used to evaluate the sensitizing effect of prolactin at a low, sublethal concentration. After 24 h, the cells were detached using trypsin and stained with 2:100 propidium iodide (556,463, BD) and 3:100 annexin V (556,419, BD) in HBSS for 15 min. Samples were analyzed in the flow cytometer using the Red-B laser to identify propidium iodide and Green-B laser to identify annexin V. No stain and single stain controls were used for gating and compensation. The experiments were repeated without the PRL pretreatment.

Next, the experiments were repeated to incorporate fluid shear stress during the TRAIL exposure step in the cells with and without prolactin treatment. After the 4-day PRL treatment, the cells were detached with trypsin, and the TRAIL treatments were added. Cone-and-plate viscometers (Brookfield) equipped with a CP-41 spindle were blocked with 5% BSA (A1470-100G, Sigma Aldrich) in HBSS with Ca^2+^ and Mg^2+^. The cells were sheared at 188 s^−1^ for 30 min at RT and then plated into a fresh 24-well plate [47]. After 24 h, an annexin V–propidium iodide assay was carried out as described above. TRAIL sensitization was calculated by subtracting the cell viability of the tested group by the cell viability of the control group (0 ng/mL TRAIL in static conditions) divided by the cell viability of the control group (Eq. 5). Data are available in Additional File 3: Data S3.

5 $$TRAIL sensitization=\frac{\%Cells,control - \%Cells}{\%Cells,control}$$

### Dual affinity liposome assays

The dual affinity liposomes were synthesized following the protocol described in the publication by *Lopez-Cavestany *et al*. (2023)* [26]. The experimental setup using peripheral blood from healthy volunteers was replicated. The control TRAIL concentration was 590 ng/mL, matching the dosage of the liposome treatments. Protocols involving human subjects were previously approved by the Institutional Review Board at Vanderbilt University, and informed consent was obtained from all healthy volunteers in the study. For quantification of cell death, leukocytes and cancer cells were separated via gradient centrifugation using one-step polymorphs (AN221725, Accurate Chemical). The buffy coat was washed once in HBSS and centrifuged at 300 × g for 5 min. The cell pellet was then resuspended in complete media and incubated for 24 h. The following day, the cells were lifted using trypsin, and the cancer cell population was enriched via negative selection in a magnetic column. All of the cells were resuspended in 80 µL of MACS buffer (130–091–376, Miltenyi Biotec) and 20 µL of anti-CD45 magnetic beads (130–045–801, Miltenyi Biotec) and then incubated for 15 min at 4 °C before passing through a magnetic column (130,042,201, Miltenyi Biotec). Cell viability was quantified via flow cytometry using an annexin V and propidium iodide assay. Any remaining immune cells after the separation were labeled with an anti-CD45 antibody pre-conjugated with the fluorophore eFluor450 (48–0459-42, Thermo Scientific) at 1:100 in 5% BSA for 30 min. This allowed them to be excluded from the analysis of cell viability. The cell population was then stained with annexin V (556,419, BD) at 2:100 and propidium iodide (556,463, BD) at 3:100 for 15 min. During flow cytometry, the Red-B laser to identify propidium iodide, Green-B laser to identify annexin V, and Blue-V laser to gate out the remaining immune cell population (CD45 + cells) were used. No stain and single stain controls were used for gating and compensation. Data are available in Additional File 3: Data S3.

### Quantification and statistical analysis

All values are plotted as mean ± SD. Experiments were repeated to obtain three independent replicates for each condition. Outliers were identified using a ROUT’s test with *Q* = 0.5% and removed. Data analysis was performed on GraphPad Prism using ordinary one-way ANOVA or two-way ANOVA when appropriate. Linear regressions were also calculated using GraphPad Prism. Statistical significance is shown as * for *p* < 0.05, ** for *p* < 0.01, *** for *p* < 0.001, and **** for *p* < 0.0001.

## Supplementary Information

Additional file 1. Abstract in Spanish. 

Additional file 2. Fig. S1—Changes in STAT3 phosphorylation, JAG1 expression, and cleaved Notch1 levels over time with PRL treatment assessed by flow cytometry in COLO320 cells. Fig. S2—Changes in cancer stem cell and EMT protein expression over time with PRL treatment assessed by flow cytometry in COLO320 cells. Fig. S3—Model fit to in vitro data with initial protein expression values and kinetic constants. Fig. S4—Computational results from the first set of Monte Carlo simulations. Fig. S5—Computational results from the second set of Monte Carlo simulations. Fig. S6—Results from tolerance levels of fitted parameter values. Fig. S7—Computational results from the sensitivity analysis. Table S1—Initial computational model values for protein expression. Table S2—Initial computational model values for kinetic constants following the numbering in the code and schematic drawing of the signaling cascade (red). Table S3—Fitted parameters from iteration 7418 of the first set of Monte Carlo simulations and iteration 21455 of the second set of Monte Carlo simulations.

Additional file 3. Data S1 – HT29 protein expression quantification after treatment with pregnancy-level PRL concentrations. Data S2 – COLO320 protein expression quantification after treatment with pregnancy-level PRL concentrations. Data S3 – Cell viability quantification of non-treated and PRL-treated HT29 cells under FSS after soluble TRAIL, and liposomal TRAIL treatments.

## Data Availability

All data generated or analyzed during this study are included in this published article and its supplementary information files. The MATLAB code and computational data files can be found on Zenodo (10.5281/zenodo.16639924).[46] Further information and requests for resources and reagents should be directed to and will be addressed by the lead contact, Dr. Michael King (mk182@rice.edu).

## Abbreviations

CRC: Colorectal cancer
PRL: Prolactin
EMT: Epithelial-to-mesenchymal transition
PRLR: Prolactin receptor
JAK: Janus kinase
STAT: Signal transducer and activator of transcription
JAG1: Jagged 1
NICD: Notch1 intracellular cleaved domain
TRAIL: Tumor necrosis factor-related apoptosis inducing ligand
CTC: Circulating tumor cell
MFI: Mean fluorescent intensity
RMSE: Root-mean-square error
FSS: Fluid shear stress
DA: Dual affinity
CSV: Cell-surface vimentin
ES: E-selectin
EOCRC: Early-onset colorectal cancer
PHI: Protected health information
ODE: Ordinary differential equation
