# Supplementary material for: Increased prolactin levels in pregnancy affect colorectal cancer aggressiveness
Source: BMC Biol. 2026 Jan 7;24:19. doi: 10.1186/s12915-025-02500-8 (PMC12825205; doi:10.1186/s12915-025-02500-8)
Supplement: Supplementary file 1 — Additional file 1. Abstract in Spanish. [file 12915_2025_2500_MOESM1_ESM.docx]

Additional File 1

**Increased Prolactin Levels in Pregnancy Affect Colorectal Cancer Aggressiveness**

M. Lopez-Cavestany, O.A. Wright, A.T. Carter, B. O’Brian, C. Eng, M.R. King

This document contains:

Abstract in Spanish

*Publisher’s note:*

*This translation in Spanish was submitted by the authors and we reproduce it as supplied. It has not been peer reviewed. Our editorial processes have only been applied to the original abstract in English, which should serve as reference for this article. This translated abstract is published under the same licence as the article.*

*Resumen:* El cáncer colorrectal (CRC) se diagnostica durante aproximadamente 1 de cada 13.000 embarazos y se asocia con peores pronósticos, incluyendo una mayor incidencia de metastásis en el momento del diagnóstico y una menor supervivencia en comparación con pacientes no embarazadas. En este estudio investigamos dos factores clave que contribuyen a este fenómeno: (1) el aumento de la agresividad de las células cancerígenas causado por los niveles elevados de prolactina (PRL) durante el embarazo, y (2) las limitaciones de las opciones terapéuticas disponibles para pacientes embarazadas con CRC.

*Resultados:* Por primera vez demostramos que los niveles de PRL durante el embarazo aumentan la señalización JAK2/STAT3 y JAG1/NOTCH1 en células de CRC, incrementando la transición epitelio-mesénquima (EMT) y la expresión de proteínas asociadas a un fenotipo de células madre cancerosas. Desarrollamos y ajustamos un modelo in silico de la vía de señalización JAK2/STAT3 basado en nuestros datos in vitro, identificando nodos específicos dentro de la cascada que son especialmente sensibles a las fluctuaciones de PRL durante el embarazo. Clínicamente, destacamos datos de casos de CRC del Vanderbilt University Medical Center, que muestran un estadío más avanzado en el diagnóstico en pacientes embarazadas y las opciones terapéuticas restringidas debido a preocupaciones sobre la seguridad fetal. Además, mostramos que la exposición a PRL sensibiliza a las células de CRC a la apoptosis inducida por TRAIL, lo que respalda el potencial de terapias basadas en TRAIL, particularmente en liposomas, como un enfoque terapéutico compatible con el embarazo.

*Conclusiones:* Este estudio proporciona el primer vínculo mecanístico entre los niveles de prolactina durante el embarazo y el aumento de la agresividad del CRC a través de la señalización JAK2/STAT3 y JAG1/NOTCH1. También sugerimos una nueva dirección terapéutica al demostrar que la PRL sensibiliza las células de CRC a la apoptosis inducida por TRAIL. En conjunto, el estudio subraya la necesidad de nuevas estrategias terapéuticas para el tratamiento seguro y eficaz del CRC en pacientes embarazadas.
