# Supplementary material for: Increased prolactin levels in pregnancy affect colorectal cancer aggressiveness
Source: BMC Biol. 2026 Jan 7;24:19. doi: 10.1186/s12915-025-02500-8 (PMC12825205; doi:10.1186/s12915-025-02500-8)
Supplement: Supplementary file 2 — Additional file 2. Fig. S1—Changes in STAT3 phosphorylation, JAG1 expression, and cleaved Notch1 levels over time with PRL treatment assessed by flow cytometry in COLO320 cells. Fig. S2—Changes in cancer stem cell and EMT protein expression over time with PRL treatment assessed by flow cytometry in COLO320 cells. Fig. S3—Model fit to in vitro data with initial protein expression values and kinetic constants. Fig. S4—Computational results from the first set of Monte Carlo simulations. Fig. S5—Computational results from the second set of Monte Carlo simulations. Fig. S6—Results from tolerance levels of fitted parameter values. Fig. S7—Computational results from the sensitivity analysis. Table S1—Initial computational model values for protein expression. Table S2—Initial computational model values for kinetic constants following the numbering in the code and schematic drawing of the signaling cascade (red). Table S3—Fitted parameters from iteration 7418 of the first set of Monte Carlo simulations and iteration 21455 of the second set of Monte Carlo simulations. [file 12915_2025_2500_MOESM2_ESM.docx]

Additional File 2

**Increased Prolactin Levels in Pregnancy Affect Colorectal Cancer Aggressiveness**

M. Lopez-Cavestany, O.A. Wright, A.T. Carter, B. O’Brian, C. Eng, M.R. King

This document contains:

Fig. S1-S7

Tables S1-S3


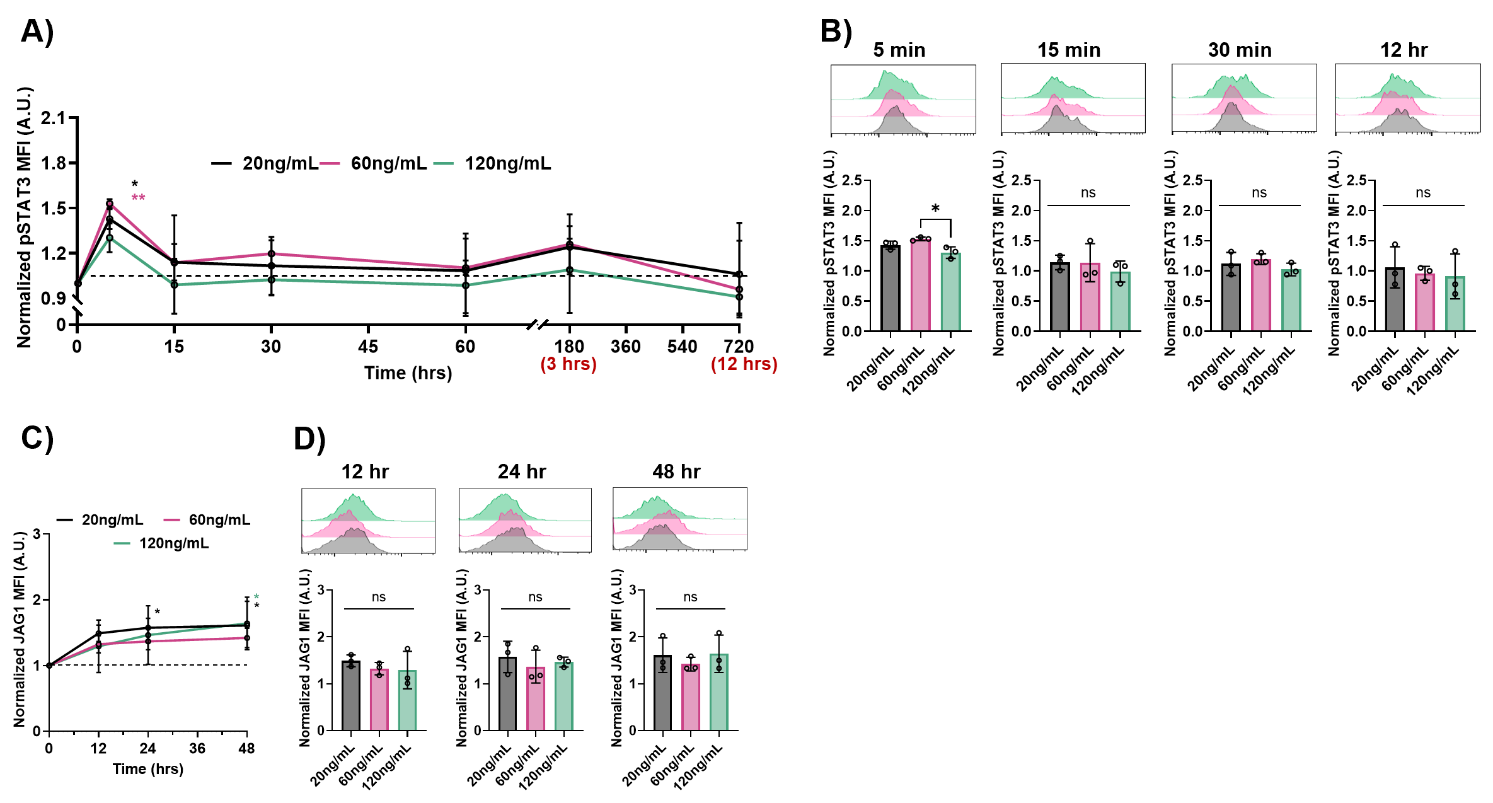


**Fig. S1. Changes in STAT3 phosphorylation, JAG1 expression, and cleaved Notch1 levels over time with PRL treatment assessed by flow cytometry in COLO320 cells.**

**A)** XY plot of pSTAT3 expression from 0 to 12 hr treated with 20 ng/mL, 60 ng/mL, and 120 ng/mL of PRL. **B)** FC histogram and quantification of pSTAT3 expression at 5 min, 15 min, 30 min, and 12 hr post-treatment. **C)** XY plot of JAG1 expression from 0 to 48 hr treated with 20 ng/mL, 60 ng/mL, and 120 ng/mL of PRL. **D)** FC histogram and quantification of JAG1 expression at 12, 24, and 48 hr post-treatment. Statistical significance was evaluated by two-way ANOVA or ordinary one-way ANOVA, and is shown as * p<0.05, ** p<0.01, *** p<0.001, and **** p<0.0001 (n=3 biological replicates).

**
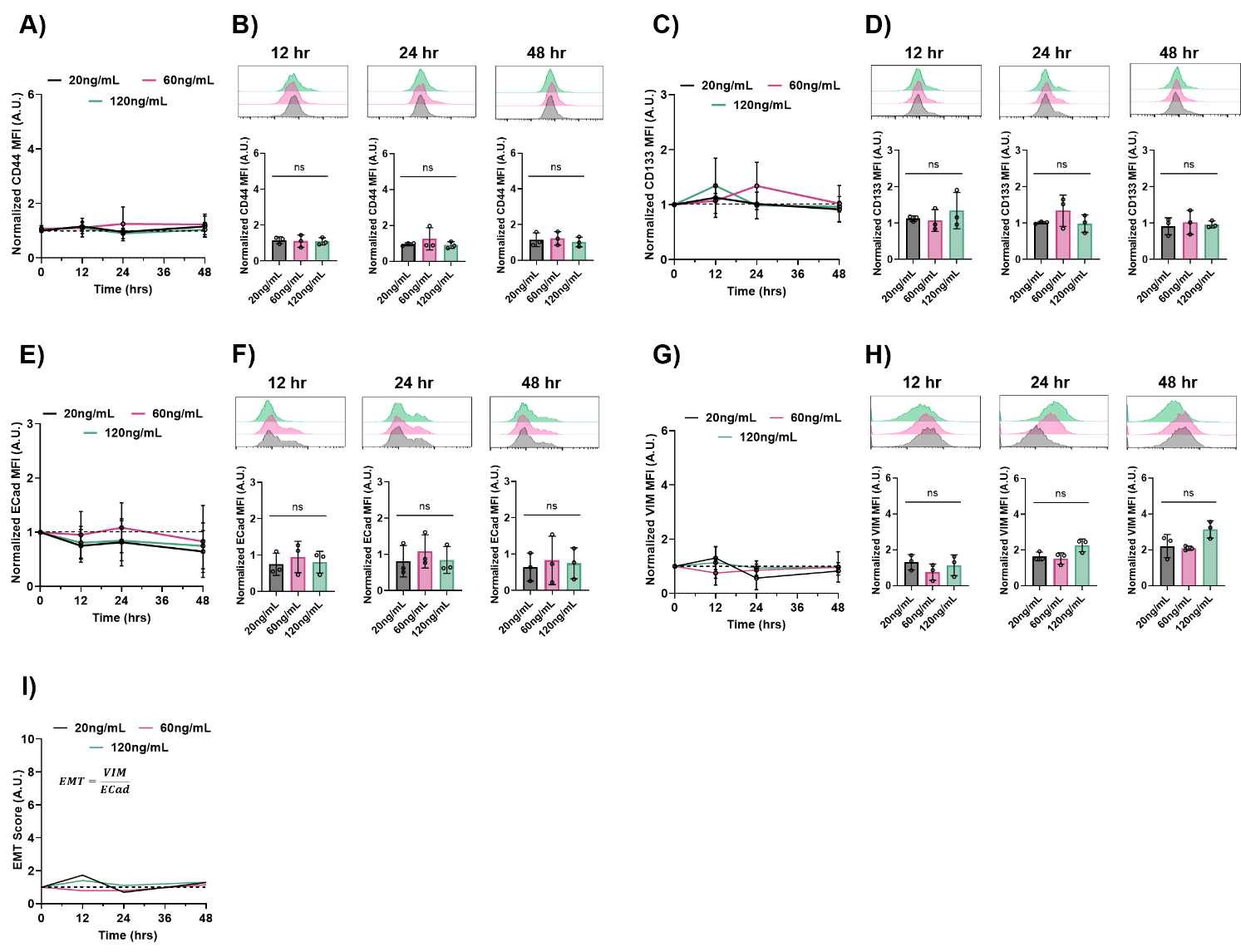
**

**Fig. S2. Changes in cancer stem cell and EMT protein expression over time with PRL treatment assessed by flow cytometry in COLO320 cells.**

**A)** XY plot of CD44 expression from 0 to 48 hr treated with 20 ng/mL, 60 ng/mL, and 120 ng/mL of PRL. **B)** FC histogram and quantification of CD44 expression at 12, 24, and 48 hr post-treatment. **C)** XY plot of CD133 expression from 0 to 48 hr treated with 20 ng/mL, 60 ng/mL, and 120 ng/mL of PRL. **D)** FC histogram and quantification of CD133 expression at 12, 24, and 48 hr post-treatment. **E)** XY plot of E-cadherin expression from 0 to 48hr treated with 20 ng/mL, 60 ng/mL, and 120 ng/mL of PRL. **F)** FC histogram and quantification of E-cadherin expression at 12, 24, and 48 hr post-treatment. **G)** XY plot of vimentin expression from 0 to 48hr treated with 20 ng/mL, 60 ng/mL, and 120 ng/mL of PRL. **H)** FC histogram and quantification of vimentin expression at 12, 24, and 48 hr post-treatment. Statistical significance was evaluated by two-way ANOVA or ordinary one-way ANOVA, and is shown as * p<0.05, ** p<0.01, *** p<0.001, and **** p<0.0001 (n=3 biological replicates).


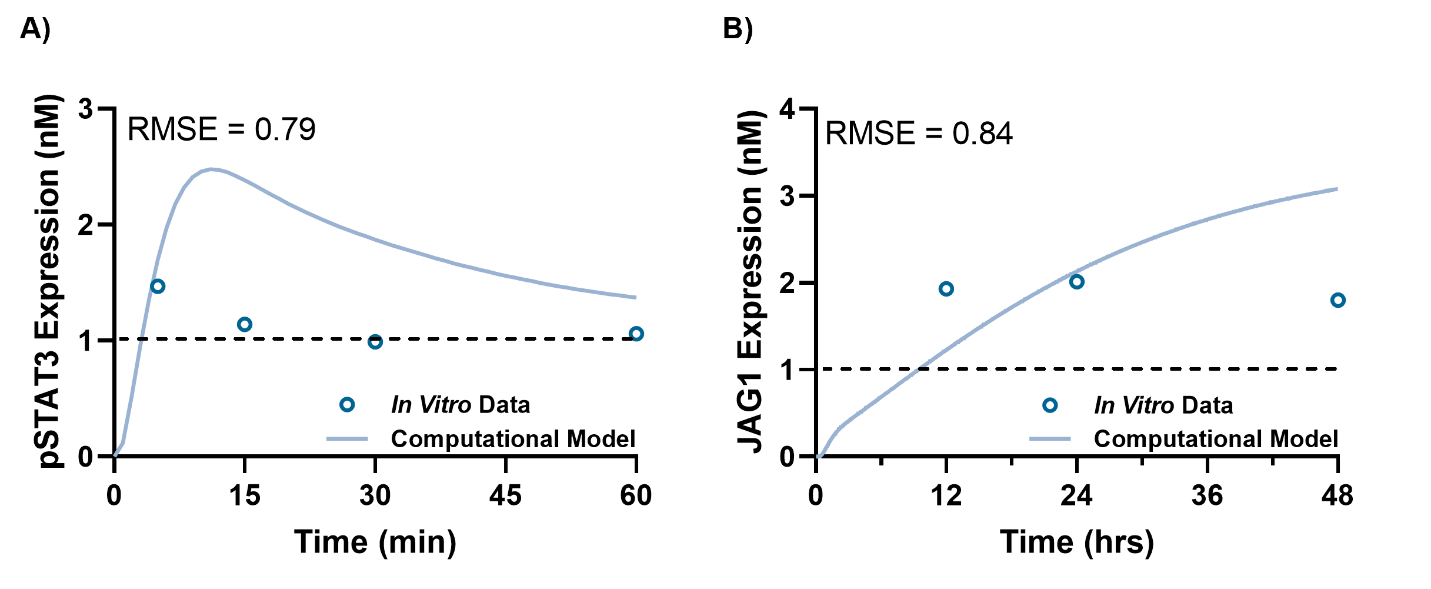


**Fig. S3. Model fit to *in vitro* data with initial protein expression values and kinetic constants.**

Model fit using the initial conditions from the Mortlock et al. study and 120 ng/mL of PRL as the treatment to **A)** the mean *in vitro* pSTAT3 value after treatment with 120 ng/mL in the HT29 cells, and **B)** the mean *in vitro* JAG1 value after treatment with 120 ng/mL in the HT29 cells.

**
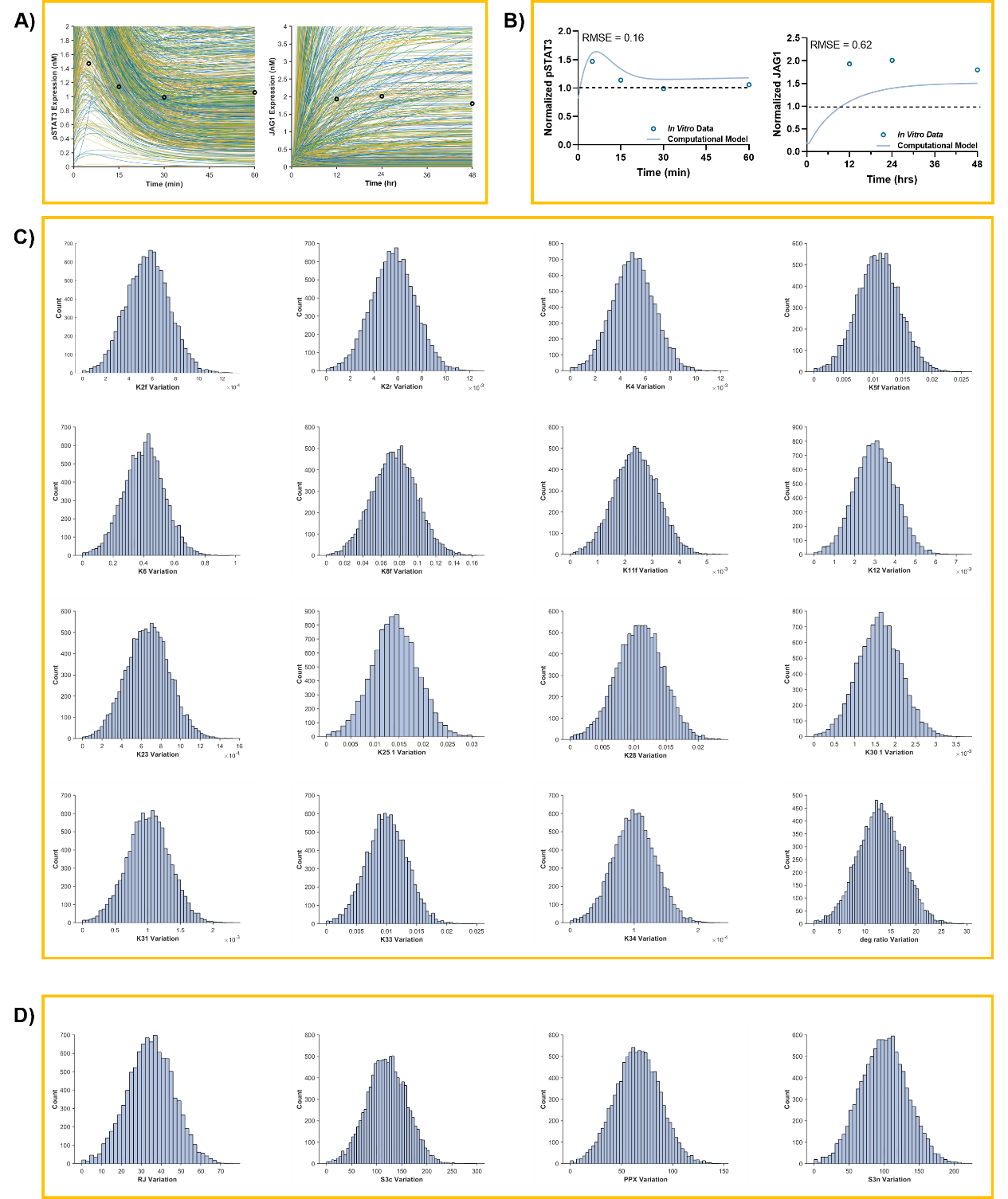
**

**Fig. S4. Computational results from the first set of Monte Carlo simulations.**

**A)** Plots of 1000 randomly selected iterations of the pSTAT3 and JAG1 concentration curves from the Monte Carlo simulations. **B)** Plots of the best fit curves for pSTAT3 and JAG1 expression to the *in vitro* data, corresponding to iteration number 7418. **C)** Histograms of the distribution of each of the randomly varied kinetic constants used in the 10,000 iterations of the model. **D)** Histograms of the distribution of each of the randomly varied protein initial conditions used in the 10,000 iterations of the model.


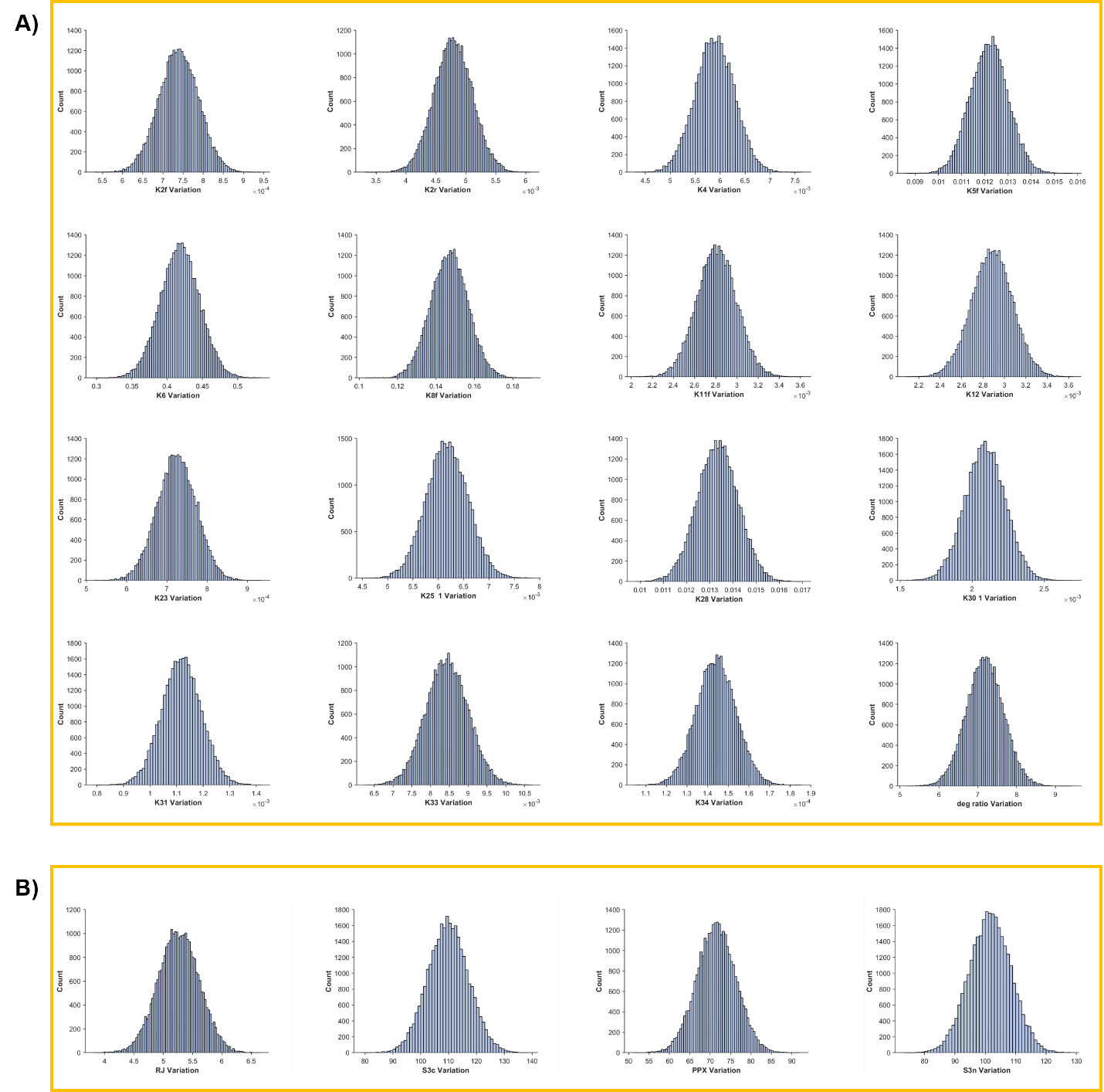


**Fig. S5. Computational results from the second set of Monte Carlo simulations.**

**A)** Histograms of the distribution of each of the randomly varied kinetic constants used in the 30,000 iterations of the model. **B)** Histograms of the distribution of each of the randomly varied protein initial conditions used in the 30,000 iterations of the model.

**
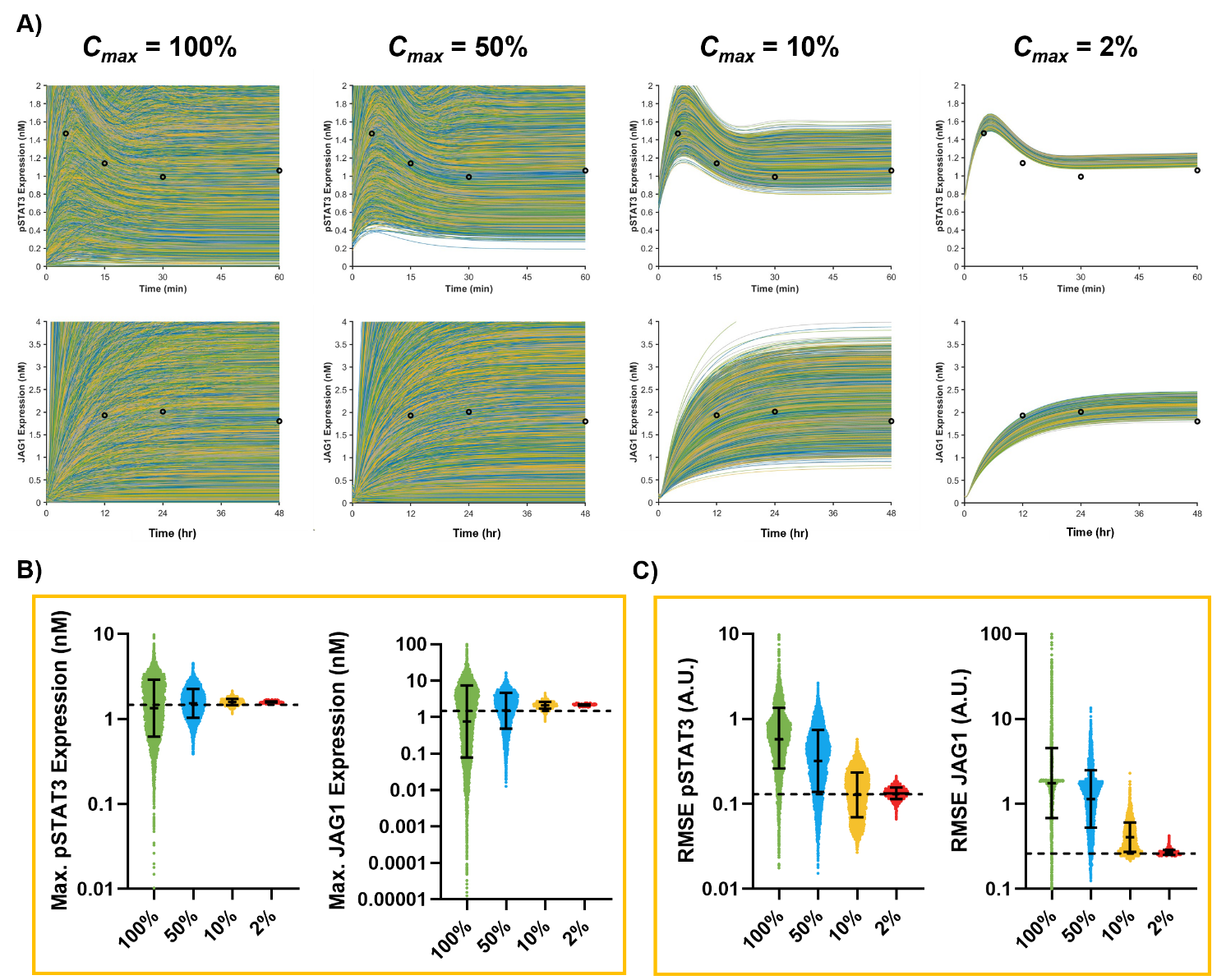
**

**Fig. S6. Results from tolerance levels of fitted parameter values.**

**A)** Plots of the pSTAT3 and JAG1 concentration curves from the Monte Carlo simulations where C_max_ is set to 50%, 10%, and 2%. **B)** Comparison of maximum pSTAT3 values and RMSE for 10,000 model iterations between the 3 C_max_ values. **C)** Comparison of maximum JAG1 values and RMSE for 10,000 model iterations between the 3 C_max_ values. Dotted lines represent the maximum values and calculated RMSE for the fitted model before the perturbations.

**
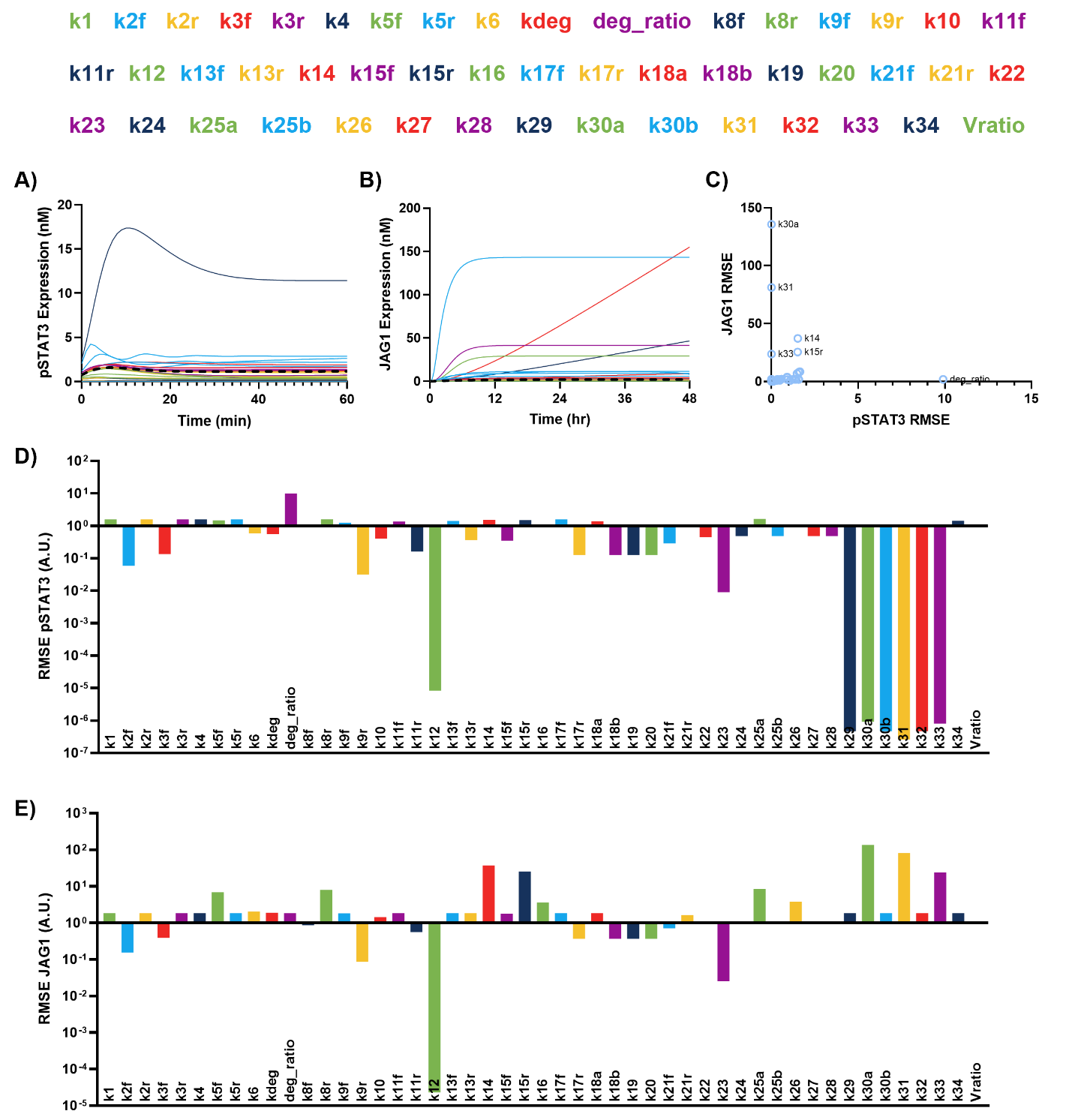
**

**Fig. S7. Computational results from the sensitivity analysis.**

Fluctuations in **A)** pSTAT3 and **B)** JAG1 expression during the sensitivity analysis using the computational model with the final fitted parameters where each line represents one kinetic constant set to zero. **C)** XY scatter of RMSE values for pSTAT3 and JAG1 comparing the effects of zeroing each kinetic constant. **D)** Plot of RMSE of pSTAT3 compared to the baseline computational model using the fitted kinetic constants. **E)** Plot of RMSE of pSTAT3 compared to the baseline computational model using the fitted kinetic constants.

**Table S1. Initial computational model values for protein expression.**

| Protein | Initial Value^1,2^ | Reference |
| --- | --- | --- |
| PRL | 5.454^3^ | (Biswas & Rodeck, 1976) |
| RJ | 34.8259062 | (Mortlock et al., 2020) |
| S3c | 120 | (Yamada et al., 2003) |
| SHP2 | 78.2373679 | (Mortlock et al., 2020) |
| PPX | 66.0051475 | (Mortlock et al., 2020) |
| PPN | 143.870996 | (Mortlock et al., 2020) |
| S3n | 100 | (Yamada et al., 2003) |

^1^All proteins not listed were assigned an initial value of zero.

^2^Units for initial values are in nanomolar (nM).

^3^This value is equivalent to 120 ng/mL.

**Table S2. Initial computational model values for kinetic constants following the numbering in the code and schematic drawing of the signaling cascade (red).**

| Constant | Initial Value^1^ | Reference |  | Constant | Initial Value | Reference |
| --- | --- | --- | --- | --- | --- | --- |
| k1 | 3.08E-03 | (Mortlock et al., 2020) |  | k17 | 3.55E-02 | (Mortlock et al., 2020) |
| k2f | 5.60E-05 | (Brelje et al., 2002) |  | k17r | 0.062303131 | (Brelje et al., 2002) |
| k2r | 5.60E-03 | (Brelje et al., 2002) |  | k18a | 1.00E-02 | (Brelje et al., 2002) |
| k3 | 0.063415984 | (Brelje et al., 2002) |  | k18b | 4.00E+02 | (Brelje et al., 2002) |
| k3r | 0.2 | (Brelje et al., 2002) |  | k19 | 0.001603988 | (Brelje et al., 2002) |
| k4 | 5.00E-03 | (Brelje et al., 2002) |  | k20 | 1.00E-02 | (Brelje et al., 2002) |
| k5 | 0.0108715 | (Brelje et al., 2002) |  | k21 | 0.052168498 | (Brelje et al., 2002) |
| k5r | 8.00E-01 | (Brelje et al., 2002) |  | k21r | 1.00E-01 | (Brelje et al., 2002) |
| k6 | 4.00E-01 | (Brelje et al., 2002) |  | k22 | 0.000506751 | (Brelje et al., 2002) |
| kdeg | 2.57E-04 | (Yamada et al., 2003) |  | k23 | 0.000665214 | (Brelje et al., 2002) |
| deg_ratio | 13.07281945 | (Mortlock et al., 2020) |  | k24 | 0.00124969 | (Mortlock et al., 2020) |
| k8 | 0.074430614 | (Brelje et al., 2002) |  | k25a | 0.014087273 | (Brelje et al., 2002) |
| k8r | 1.00E-01 | (Brelje et al., 2002) |  | k25b | 4.00E+02 | (Brelje et al., 2002) |
| k9 | 0.000879762 | (Brelje et al., 2002) |  | k26 | 1.00E-03 | (Brelje et al., 2002) |
| k9r | 2.00E-01 | (Brelje et al., 2002) |  | k27 | 6.12582E-05 | (Brelje et al., 2002) |
| k10 | 3.00E-03 | (Brelje et al., 2002) |  | k28 | 0.010887257 | (Brelje et al., 2002) |
| k11 | 0.002400238 | (Brelje et al., 2002) |  | k29 | 1.00E-02 | (Mortlock et al., 2020) |
| k11r | 2.00E-01 | (Brelje et al., 2002) |  | k30a | 0.001614721 | (Mortlock et al., 2020) |
| k12 | 3.00E-03 | (Brelje et al., 2002) |  | k30b | 4.00E+02 | (Mortlock et al., 2020) |
| k13 | 2.00E-07 | (Brelje et al., 2002) |  | k31 | 1.00E-03 | (Mortlock et al., 2020) |
| k13r | 0.225430866 | (Brelje et al., 2002) |  | k32 | 5.00E-04 | (Mortlock et al., 2020) |
| k14 | 0.005886461 | (Brelje et al., 2002) |  | k33 | 1.00E-02 | (Mortlock et al., 2020) |
| k15 | 0.001460885 | (Brelje et al., 2002) |  | k34 | 1.93E-05 | (Merino et al., 1994) |
| k15r | 2.00E-01 | (Brelje et al., 2002) |  | Vratio | 5.00E-01 | (Yamada et al., 2003) |
| k16 | 0.0145287 | (Brelje et al., 2002) |  |  |  |  |

^1^Rate constants are shown as nM/s

**Table S3. Fitted parameters from iteration 7418 of the first set of Monte Carlo simulations and iteration 21455 of the second set of Monte Carlo simulations.**

| Parameter^1^ | MC1 Fitted Value^2^ | MC2 Fitted Value^2^ |
| --- | --- | --- |
| RJ | 29.6141 | 5.0802 |
| PPX | 67.2871 | 70.3604 |
| S3c | 117.5831 | 117.1798 |
| S3n | 88.6565 | 101.8617 |
| JAG | 0.2015 | 0.1528 |
| k2f | 6.02E-05 | 7.7971E-04 |
| k2r | 0.0077 | 0.005 |
| k4 | 0.0054 | 0.0052 |
| k5f | 0.0080 | 0.0124 |
| k6 | 0.5411 | 0.4417 |
| k8f | 0.0973 | 0.1814 |
| k11f | 0.0016 | 0.0028 |
| k12 | 0.0038 | 0.0031 |
| k23 | 0.0007 | 6.8485E-04 |
| k25_1 | 0.01769 | 0.0056 |
| k28 | 0.0128 | 0.0132 |
| k30_1 | 0.0034 | 0.0021 |
| k31 | 0.0013 | 0.0011 |
| k33 | 0.0167 | 0.0099 |
| k34 | 8.88E-06 | 1.4518E-04 |
| deg_ratio | 14.0308 | 7.1442 |

^1^All proteins and kinetic constants not listed remained the same.

^2^Units for initial values are in nanomolar (nM) and rate constants are shown as nM/s.
